# Theoretical framework and reliability verification of the loaded three-zone structural model in coal mine overburden strata

**DOI:** 10.1038/s41598-025-19335-6

**Published:** 2025-10-09

**Authors:** Yi Liu, Quanjie Zhu, Quande Wei, Guangyu Yang, Yichao Zhang, Qilin Hao, Dongsheng Jiang

**Affiliations:** 1 Information Institute of Ministry of Emergency Management, Beijing, 100029 China; 2 School of Emergency Technology and Management, North China Institute of Science and Technology, Sanhe, 065201 Hebei China; 3 China Coal Mining Research Institute Co., Ltd., Beijing, 100013 China; 4 Coal Mining & Designing Department, Tiandi Science & Technology Co., Ltd., Beijing, 100013 China; 5 China Coal Technology & Engineering Xi’an Research Institute (Group) Co., Ltd., Xi’an, 710077 China; 6 Yima Coal Industry Group Corporation Ltd., Yima, 472300 China

**Keywords:** Rock burst, Roof structure, Abutment pressure, The three zone structureloading model, Rock burst hazard, Mechanical engineering, Seismology

## Abstract

The fundamental stresses that determine rock burst in coal mine workings are primarily gravitational stress and tectonic stress, with the inducing force mainly coming from the stress generated by the structural movement of the overlying strata in the workings. The variable structural forms and movement patterns of the overlying strata are the main reasons for the complex mechanisms of rock burst and the various forms of impacts. By studying the relationship between the boundary conditions of the workings and the structure of the overlying strata, and based on mine pressure and strata control theory, a ‘Three Load Zones’ structural model of the overlying strata influencing the stress field of rock burst in the workings is proposed. The definition of the Three Load Zones and the subsequent computational model are introduced, and the differences between the ‘Three Load Zones’ and the traditional ‘Three Zones’ are explained. Using microseismic monitoring technology, the evolutionary characteristics of the overlying strata under mining conditions are studied, and the evolutionary patterns of the Three Load Zones are quantitatively analyzed. The influence of the ‘Three Load Zones’ on lateral and strike support pressure in the working face is also discussed. An estimation model for the ‘Three Load Zones’ stress is proposed, and the application of this theory in evaluating rock burst in the workings is analyzed. Through field measurements and applications, combined with theoretical analysis, microseismic monitoring, and numerical simulation methods, the validity of the proposed ‘Three Load Zones’ theory is demonstrated. This method can be used for the risk assessment and classification of rock burst in coal mine workings, providing a theoretical basis for targeted mitigation of rock burst.

## Introduction

The occurrence of rock burst is primarily influenced by the combined effects of gravitational stress, tectonic stress, and mining-induced stress, with gravitational stress and mining-induced stress playing a dominant role in deep mining^1,2^. Gravitational stress is determined by the mining depth, while the structural type and movement patterns of the overlying strata are the root causes of mining-induced stress. Therefore, studying the relationship between the structure of the overlying strata and mining-induced stress, establishing a structural model of the overlying strata that includes mining depth and mining-induced factors, and proposing a stress calculation model for evaluating rock burst risk is a crucial theoretical topic in the prevention and control of rock burst.

Both domestic and international scholars have conducted extensive research on the mechanisms and prediction of rock burst. However, due to the complexity and uncertainty of the overlying strata, the occurrence of rock burst is highly random and localized, presenting numerous challenges. In recent years, researchers have explored the triggering mechanisms and stress field distribution of rock burst through microseismic monitoring technology and numerical simulation methods^3–5^. Based on this, various strata structural models have been proposed, aiming to provide theoretical support for the prediction and prevention of rock burst through accurate stress field estimation and analysis of evolution patterns.

While there has been extensive research on the spatial structure of overlying strata in mining operations, the distribution of mining-induced stress, and the classification and risk evaluation of rock burst, there is relatively little research that focuses on the relationship between overlying strata structure^6–11^ mining-induced stress^12,13^, rock burst types, and rock burst risk^14–16^, particularly in terms of classification and targeted mitigation. The types and conditions of rock burst vary across mines, and due to the unclear understanding of the mechanisms of rock burst, mining enterprises often adopt a one-size-fits-all and crude approach to control rock burst, such as ‘the section can only be larger, not smaller; instruments can only be placed higher, not lower; supports can only be stronger, not weaker; measures can only be increased, not decreased.’ Under this guiding philosophy, normal mining activities are severely restricted, leading to a waste of human and material resources without achieving satisfactory results.

Foreign scholars began researching the theories related to rock burst as early as the 1980s. Experts generally believe that the occurrence of rock burst is not only related to the physical and mechanical properties of the coal seam but also closely linked to the movement patterns of the strata, emphasizing the direct impact of the deformation of the overlying strata on rock burst^7,14^. In China, research on rock burst has primarily focused on the relationship between strata structure and stress fields^17–19^. In China, research on rock burst has mainly focused on the relationship between strata structure and stress fields. In recent years, with the development of microseismic monitoring technology, scholars have introduced real-time monitoring and data analysis methods in mines. The application of microseismic monitoring technology in rock burst research has provided new directions for mine pressure analysis^20^. By real-time monitoring of microseismic events such as strata deformation, crack propagation, and energy release, researchers are able to dynamically capture changes in the stress field of the workings, thus predicting the occurrence of rock burst^21^. Reference^22^ provided an application of geomechanics in underground mining, including the causes, theoretical analysis, and prevention measures for rock burst. The book discusses, with real-world case studies, the interaction between strata structure and stress fields in mining operations. Reference^23^ focused on the role of microseismic monitoring in predicting rock burst in mines, analyzing the impact of stress changes in different strata on rock burst using actual data. Using numerical simulation and field monitoring data analysis^24^, they provided new theoretical evidence for predicting rock burst.

Although there has been a large body of research on rock burst, its complex mechanisms still require further exploration^25–27^. Future research could further improve the quantitative analysis of stress evolution patterns in each load zone. In addition, theoretical validation based on field data and numerical simulation methods remain important tools for enhancing the rock burst early warning capabilities.

With the increase in mining depth, the current methods no longer meet the needs of the field. Faced with complex geological conditions and diverse mining operations, to target and control rock burst, a scientific understanding of its causes is essential. The first question that must be addressed is: where does the force that triggers the rock burst come from, and what is the surrounding rock structure at the location of the rock burst? This paper conducts a series of fundamental studies around these two questions. Based on this, the paper investigates the structural types of overlying strata in the working face recovery stage under typical mining boundary conditions, and on this basis, establishes the ‘Three Load Zones’ theoretical model. It also analyzes the distribution patterns of lateral and strike support pressures exerted on the coal body around the working face under different overlying strata conditions. The research findings can be applied in the evaluation, monitoring, and prevention of rock burst in mines, providing a theoretical basis for safe mining and scientific control of rock burst in mines with potential rockburst hazards.

## Introduction of the ‘three load zones’ concept

### Overview of the working face and introduction to rock burst incidents

**Fig. 1:**
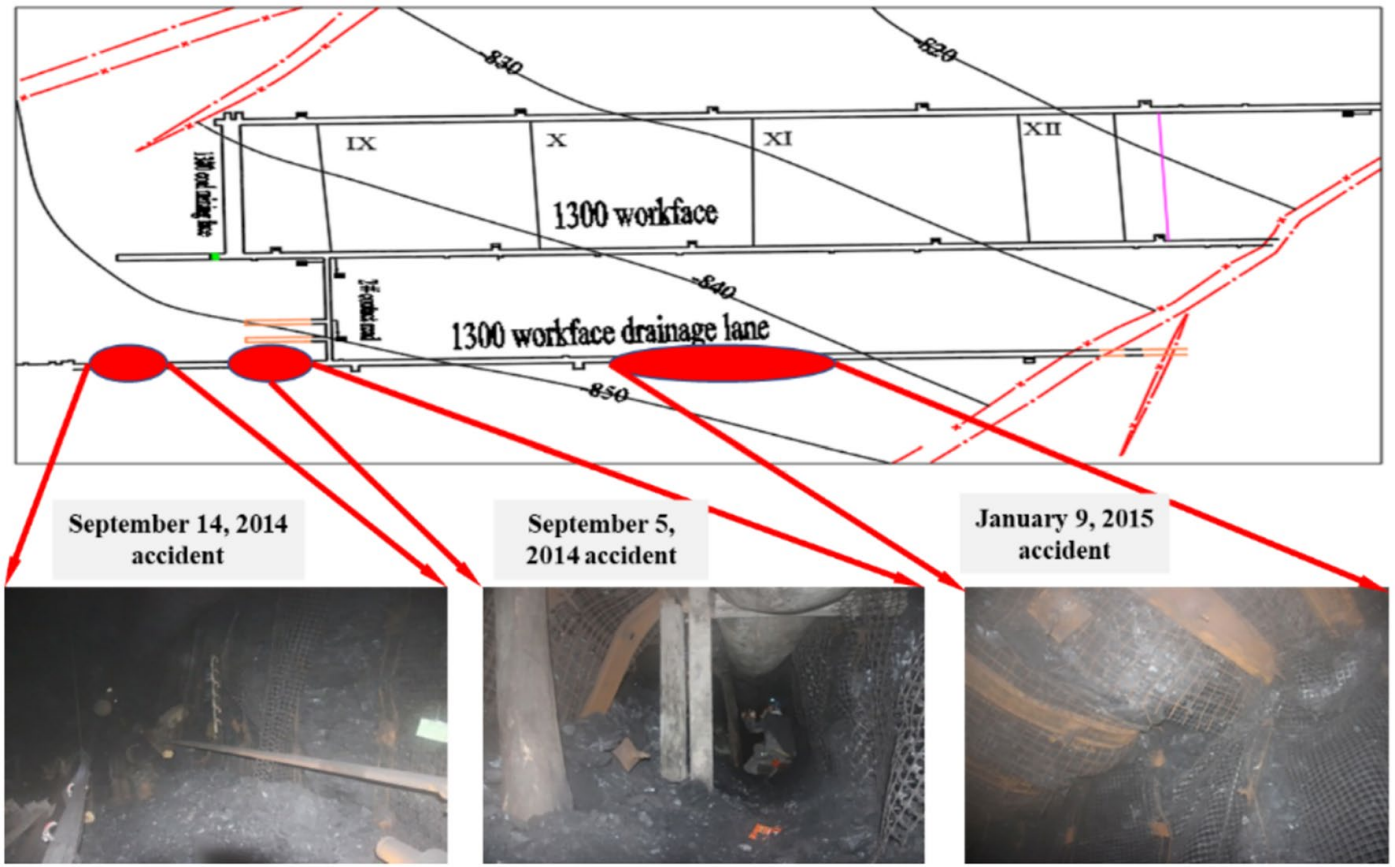
Distribution of typical rock burst events at the 1300 working face of the coal mine.

The 1300 working face at Yuncheng Coal Mine has an average burial depth of approximately 890 m, with a dip length of 100 m. It adopts a fully mechanized top-coal caving mining method and is the first mining face in the mining area. On one side of the working face is the Bailizhuang Branch No. 4 Fault, with the cut-off located in the anticlinal structural zone. To the east is the 1300 drainage roadway, with an 80 m-wide coal pillar left between the 1300 drainage roadway and the 1300 track roadway. According to the rock burst tendency report, the coal seam, roof, and floor of the 1300 working face all exhibit weak rock burst tendency. In recent years, several rock burst incidents have occurred at this working face (as shown in Fig. 1):

- On September 5, 2014, when the 1300 working face advanced about 17 m, a localized rock burst occurred 10 m north and 120 m south of the No. 2 crosscut in the 1300 working face drainage roadway, resulting in varying degrees of roof subsidence and rib squeezing within the affected area.
- On September 14, 2014, when the 1300 working face advanced about 31 m, a localized rock burst incident occurred between 90 and 140 m south of the No. 2 crosscut in the 1300 working face drainage roadway, with significant roof subsidence, rib squeezing, damage to the mesh in parts of the roof and ribs, and coal drop within a 50 m range.
- On January 9, 2015, when the 1300 working face advanced about 496 m, a rock burst occurred between 125 and 280 m north of the No. 2 crosscut in the 1300 drainage roadway. The incident caused varying degrees of rib squeezing, cracks in the double-antistatic mesh, roof subsidence and falling, and the detachment of the explosion-proof canopy in the 155 m range of the 1300 working face drainage roadway.

In recent years, multiple rock burst incidents have occurred at Yuncheng Coal Mine, characterized by long influence distances, extended impact durations, and strong intensity. The areas where rock bursts occur show no obvious mining-induced impact and are also relatively far from the goaf. These characteristics differ significantly from the traditional rock burst mechanisms, the influence range of support pressure theory, the range of strata movement, and the mining-induced impact duration. Therefore, to address this issue, the team used the rock burst incidents at Yuncheng Coal Mine as a case study and innovatively proposed the ‘Three Load Zones’ model for the overlying strata structure in the mining face. Combining mine pressure theory and the theory of overlying strata spatial structure, the team analyzed the stress conditions and spatiotemporal patterns of rock burst occurrence at Yuncheng Coal Mine, providing a theoretical basis for targeted mitigation of rock burst.

### Introduction of the ‘three load zones’ concept

The three rock burst incidents at Yuncheng Coal Mine all occurred in the 1300 drainage roadway. From a stress perspective, the basic stress conditions for the rock burst accidents in the 1300 drainage roadway include: (1) The area where the roadway is located is in the wing of an anticlinal structure, and the northern part of the roadway crosses the FY15 fault, making the area highly influenced by tectonic stress; (2) The average burial depth of the 1300 working face is approximately 890 m, and the deep mining conditions result in the coal and rock body in the mining area experiencing high gravitational stress; (3) The 1300 working face drainage roadway is in the area affected by lateral support pressure, which significantly influences the roadway due to lateral support stress.

In terms of time, the rock burst accidents in the 1300 drainage roadway can be divided into two stages. The first stage occurs at the early stage of working face advancement (before the face, with a mining distance of 15 m to 30 m), near the No. 2 crosscut in the drainage roadway. The second stage occurs at the mid-stage of working face advancement (after the face, with a mining distance of 496 m), near the FY15 fault, about 300 m behind the working face.

**Fig. 2:**
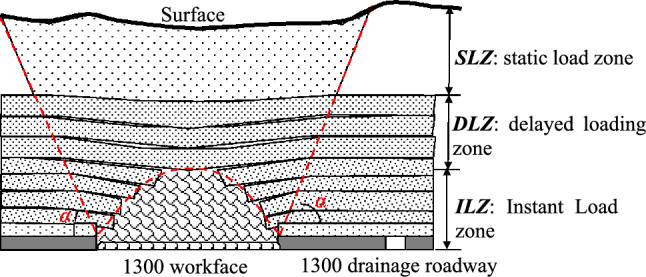
Standard structure of the three load zones in the overlying strata of the 1300 working face.

The ‘Three Load Zones’ structural model is an important innovation in the study of rock burst stress fields. This model divides the mining area into three parts: the static load zone, delayed load zone, and instantaneous load zone. By analyzing the structural features of each load zone and the movement patterns of the strata, the model deeply explores the role of different load zones in the occurrence of rock burst in the mining face. Taking Yuncheng Coal Mine as an example, it is assumed that after the working face has advanced, the three load zones develop into a standard model, as shown in Fig. 2. It can be seen that the thickness of the ‘ILZ’ zone in the 1300 working face is 60 m, the thickness of the *M*_*DLZ*_ is 280 m, and the *M*_*SLZ*_ is 550 m. By introducing the Three Load Zones model^17^ and combining the mining size conditions of the 1300 working face, the height of the three load zones in the mining area and their strata composition are determined. This can explain the stress conditions, spatiotemporal patterns, and the mechanism of the rock burst accidents in the 1300 drainage roadway.

1. Dimensions of the Three Load Zones before the working face advances: Before the working face advances, the Three Load Zones model is a degraded model. Due to the short mining distance, the movement range of the roof is also small. As a result, the Three Load Zones model undergoes degradation. The height of the ‘Instantaneous Load Zone’ (referred to as ILZ) is numerically equal to half the mining distance, and the ‘Delayed Load Zone’ (referred to as DLZ) degrades into the ‘Static Load Zone’ (referred to as SLZ) range.
2. Dimensions of the Three Load Zones after the working face advances: Due to the large mining height of the 1300 working face, the height of the ‘ILZ’ zone has already exceeded the calculated height of half the mining width. Therefore, the spatial position of the ‘DLZ’ shifts upwards. Under these conditions, the strata within the ‘DLZ’ do not fracture but instead experience delamination from the bottom upwards over an extended period of time after the working face advances. The starting position of the delamination gradually deepens along with the pressure failure of the coal body beneath it.

### Accident mechanism analysis based on the ‘three load zones’ theory

The roadway damage conditions during the occurrence of the three rock burst incidents and the Three Load Zones model are listed in Table 1. Before the working face advances, the Three Load Zones are in a degraded form, with a small impact range and low degree of roadway damage. After the Three Load Zones are fully formed, both the impact range and the degree of roadway damage significantly increase.

**Table 1:** Correspondence table of impact ground pressure accidents and load three-band state.

Date	Load three-zone pattern	Two-sided displacement/mm	Bottom drum /mm	Roof damage pattern	Roadway damage length/m
2014.9.5	Degenerate three-band structure	200	150	Roof convergence	38
2014.9.14	Degenerate three-band structure	–	–	Roof convergence	50
2015.1.9	Complete three-band structure	≥ 1000	> 2000	Roof falling	155

1. Rock Burst Before the Working Face Advances: From the plan view, it can be seen that the areas of rock burst on September 5 and September 14 occurred in the axial part of the anticline structure, where the tectonic stress concentration factor is taken as 1.5. Near the No. 2 crosscut, the roadway is in a densely packed area, with concentrated stress from the surrounding tunnels, and the stress concentration factor is taken as 1.1. The stress (*σ*) of the coal and rock body in the impacted section of the 1300 working face drainage roadway is calculated to be 36.71 MPa, and the uniaxial compressive strength (*σ*_*c*_) of the coal body is about 15 MPa. The ratio *σ*/[*σ*_*c*_] = 2.44, which is greater than 2.0. Therefore, this location meets the critical stress condition for rock burst. When the two rock burst incidents occurred in September 2014, the 1300 drainage roadway was outside the lateral influence range of the 1300 working face and was less affected by dynamic load disturbances. The cause of the accident was the combined effect of high gravitational stress, tectonic stress, and tunnel group stress, which led to coal failure and caused the rock burst.
2. Rock burst After the Working Face Advances: According to microseismic monitoring results, a high-energy microseismic event occurred on January 9, 2015, at a location 72 m laterally from the coal rib of the 1300 working face, at the peak point of lateral support pressure in the ‘DLZ’ zone. This point is in the wing of an anticline structure, where the tectonic stress concentration factor is taken as 1.2. The stress at this point is 40.13 MPa, and the ratio *σ*/[*σ*_*c*_] = 2.68, which is greater than 2.5, indicating that it meets the critical stress condition for rock burst. When the accident occurred on January 9, the horizontal distance between the working face and the microseismic event location was greater than 300 m, meaning the location was less affected by mining activities. The cause of the accident was the slow subsidence of the ‘DLZ’ zone under high gravitational and tectonic stress, leading to stress accumulation and coal pillar failure, which triggered the rock burst.

From this, it is clear that using the ‘Three Load Zones’ structural model provides a more direct way to analyze the intrinsic relationship between the overlying strata structure and the stress distribution in the mining face. This model enables the quantification of rock burst risk and prediction, which is of great significance for the prevention and control of dynamic disasters, as well as for roadway surrounding rock control^28^. In the following sections, we will introduce the concept, evolution patterns, and calculation models of the ‘Three Load Zones’ in more detail.

## Definition and characteristics of the ‘three load zones’ in the overlying strata of the mining area

Based on the research results of the team over the years, rock burst generally exhibits one of the following three spatiotemporal characteristics: (1) The location of the rock burst is relatively close to the working face coal rib, and the occurrence time is closely related to the periodic loading of the immediate roof and the main roof, showing a short cycle and frequent occurrences; (2) The location of the rock burst is relatively far from the working face coal rib, and the occurrence time is closely related to the stability time of the rock layers behind the goaf, exhibiting a long cycle along the strike direction; (3) The location of the rock burst is far from the mining working face, and the specific location is influenced by the overall layout of the mining area, with no specific periodicity for the occurrence time. Different spatiotemporal characteristics correspond to different strata structures and their movement patterns.

It is evident that the time and spatial location of rock burst occurrences are closely related to the movement of the overlying strata and their loading modes. Therefore, it is necessary to establish an overlying strata structure model based on the movement forms and loading modes of the strata. Studying the structural forms and movement patterns of the overlying strata in the working face is of great significance for the prevention and control of dynamic disasters, as well as for roadway surrounding rock control.

In the areas of overlying strata spatial structure, mining-induced stress distribution patterns, and rock burst classification and risk evaluation, domestic and international scholars have conducted extensive and in-depth research^29^. However, there has been relatively less research aimed at the classification and management of rock burst, focusing on the relationship between overlying strata structure, mining-induced stress, rock burst types, and rock burst risk. In light of this, this chapter will study the structural types of overlying strata during the recovery stage of the working face under typical mining boundary conditions. Based on this, a Three Load Zones theoretical model will be established, and the magnitude and distribution patterns of lateral and strike support pressures accumulated in the coal body around the working face will be analyzed under different overlying strata conditions.

### Definition and thickness calculation of the ‘three load zones’

**Fig. 3:**
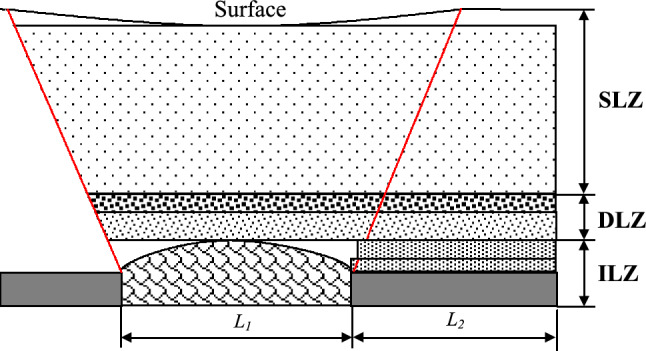
General view of three zone structure loading model.

Engineering practice has shown that, for the prevention and control of rock burst in the working face, the strata range that needs to be studied includes the immediate roof, main roof, and even the entire overlying strata up to the surface. To analyze the stress impact that the overlying strata exert on the roadway itself and its surroundings during mining operations, the entire overlying strata are divided into three zones: the ‘Instantaneous Load Zone,’ the ‘Delayed Load Zone,’ and the ‘Static Load Zone,’ collectively referred to as the ‘Three Load Zones,’ as shown in Fig. 3 (L₁ is the length of the current mining face, and L₂ is the length of the adjacent prepared mining face.). The movement of the ‘Three Load Zones’ is the main source of stress that triggers rock burst.

In addition to being influenced by the mining conditions of the working face itself, the rock burst risk of the working face is also affected by the surrounding mining conditions. Therefore, the ‘Three Load Zones’ model of the overlying strata in the mining area considers the movement of the overlying strata across the entire continuous mining range of the area. The thickness of the ‘Three Load Zones’ is considered based on the mining area as a whole, rather than a single working face. The thickness of the ‘Three Load Zones’ is jointly determined by the mining height, mining depth, and mining width of the working face.

(1) Instantaneous Load Zone (ILZ): As the recovery work progresses, the strata within this zone will experience periodic flexing, cracking, and collapse in the short term, filling the goaf immediately and forming a load-bearing structure. The thickness of this zone is closely related to the mining height. According to the definition of the ‘ILZ,’ as the working face advances from the cut-off, the overlying strata in the mining area will immediately experience collapse and intense rotational movement due to the appearance of the goaf, filling the goaf and forming a load-bearing structure. Its height *M*_*ILZ*_ can be expressed as,

1 $$M_{ILZ} = \frac{h}{{K_{A} - 1}}$$

In the equation, *h* is the mining height, and *K*_*A*_ is the swelling coefficient of the rock. The calculation method of the ‘caving zone’ in reference^30^ is used here. In the reference, the value of *K*_*A*_ typically ranges from 1.1 to 1.3. However, due to the difference in definitions between the ‘Instantaneous Load Zone’ (ILZ) and the ‘caving zone,’ the ILZ not only includes the strata that collapse as defined in reference, but also includes the strata that fracture and rotate to form a load-bearing structure in a short time as mining progresses. Therefore, the range of the ILZ is larger than that of the caving zone, and the value of *K*_*A*_ here is taken as 1.1. This shows that the height of the ILZ is closely related to the mining height of the working face, with a significant ‘heave effect’.

(2) Delayed Loading Zone (DLZ): Located above the ‘ILZ’ zone, the DLZ initially has a suspended roof in the early stage of mining. However, as the load it bears exceeds its own strength, it gradually undergoes delamination and fracture over a long period of time. For the DLZ, the stress generated by its movement will gradually become evident during the mining process and for a long time after mining is completed, until the internal strata form a stable ‘arch’ structure. Its thickness is related to the width of the goaf. As the goaf is completely filled by the strata within the ‘ILZ’ zone, higher strata, due to the lack of collapse space, cannot fracture and sink immediately. Instead, they will gradually experience delamination and fracture over a long period as the load they bear exceeds their strength. According to general strata movement theory, from the advance of the cut-off to the stage where the strata movement enters a fully mined phase, the maximum height of the fractured strata forming a structure above the mining area is approximately half the short side width (*L*) of the continuous mining range, *H*_*DLZ*_ ≈ L/2.

2 $$M_{DLZ} = H_{DLZ} - H_{ILZ} = \frac{L}{2} - 10h$$

Therefore, the short side width of the continuous mining range in the mining area determines the height of the ‘SLZ’ zone, with a significant mining width effect.

(3) Static Loading Zone (SLZ): This zone extends from above the ‘DLZ’ zone to the surface, comprising strata with good continuity. The stress exerted on the underlying rock body by this zone changes little due to mining activity. Because it is far from the mining area, the ‘SLZ’ zone is less influenced by mining activities. The overlying rock self-weight stress within the strata of the ‘SLZ’ zone has a small horizontal stress gradient, which can be considered uniformly distributed. According to the definition of the ‘SLZ,’ the strata above the ‘DLZ’ zone, extending up to the surface, form the ‘SLZ’ zone.

3 $$M_{SLZ} = H - M_{ILZ} - M_{DLZ}$$

In summary, the thickness of the ‘SLZ’ zone is determined by the mining depth H, MILZ, and MDLZ. When the working face of normal mining is under non-fully mined conditions, the strata structure follows the standard ‘ILZ’ + ’DLZ’ + ’SLZ’ three-zone model, and the thickness is calculated using the above formula. When the working face advance is very small, with L/2 < 10 h, the delayed loading effect of the overlying strata is not significant, and the ‘DLZ’ zone is considered nonexistent, causing the strata structure to degrade to a ‘ILZ’ + ’SLZ’ two-zone structure. When the working face burial depth is less than L/2, the fracture of the overlying strata will slowly extend to the surface, and the ‘SLZ’ zone completely degrades into the ‘DLZ’ range, resulting in the strata structure degrading into a ‘DLZ’ + ’ILZ’ two-zone structure. When the overlying strata have very low strength, the collapse of the strata immediately appears at the surface, and the delayed loading effect is not significant, causing the strata structure to degrade into an ‘ILZ’ + ’SLZ’ two-zone structure.

### Evolutionary patterns of the stress influence range of the ‘three load zones’

**Fig. 4:**
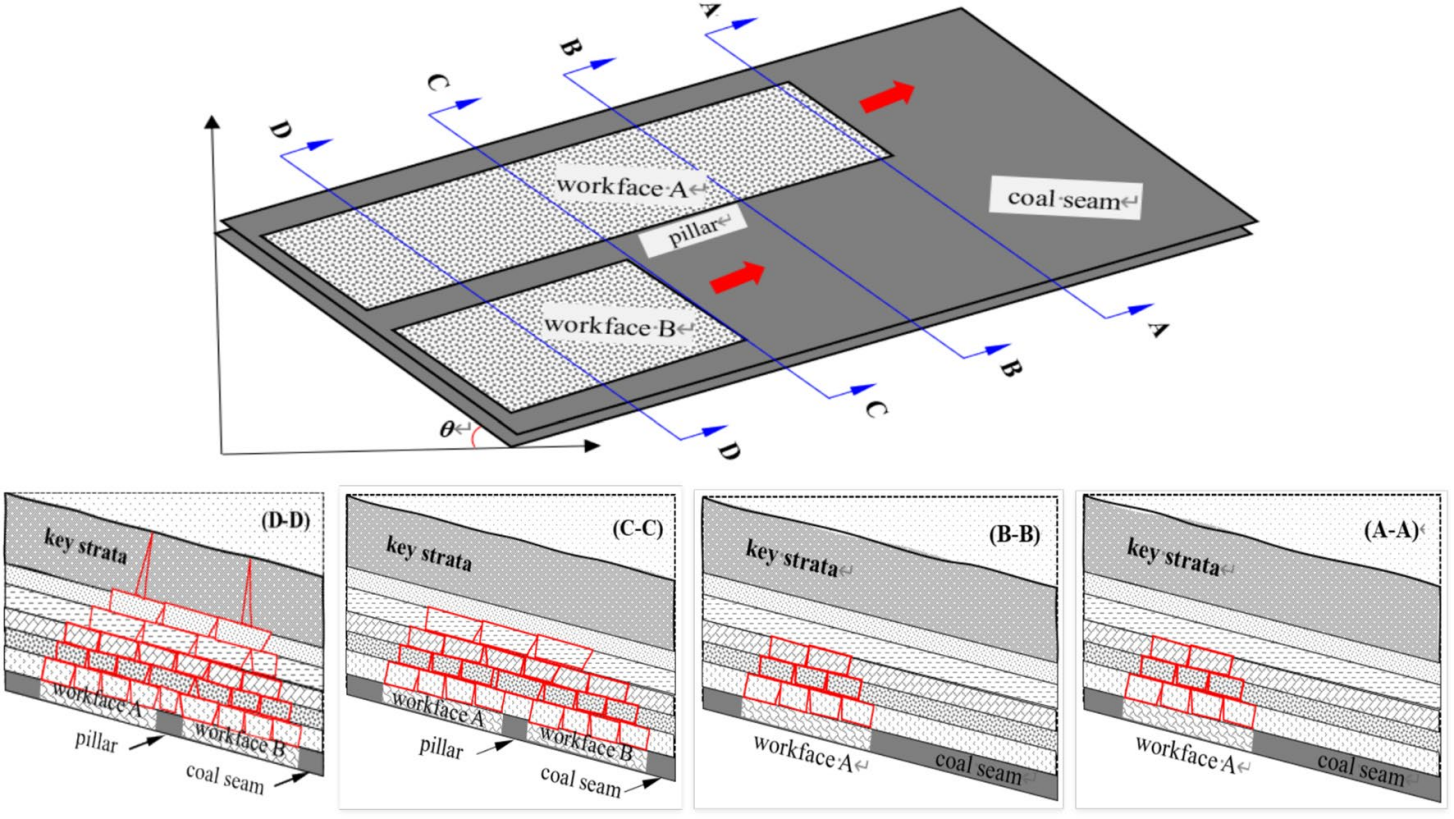
Schematic diagram of the dynamic evolution of the overlying strata spatial structure during the whole process of mining working face. (**A**) Plane diagram, (**B**) A-A profile, (**C**) B-8 profile, (**D**) C–C profile, (**E**) D-D profie.

The movement of the strata in the ‘ILZ’ and ‘DLZ’ zones of the Three Load Zones will significantly impact the stress magnitude and distribution of the underlying working face. The specific range is determined by the mining dimensions of the working face. Based on the variation in the minimum width of the goaf, the entire mining process is divided into the following stages, with the changes in the Three Load Zones analyzed separately, as shown in Fig. 4.

**Fig. 5:**
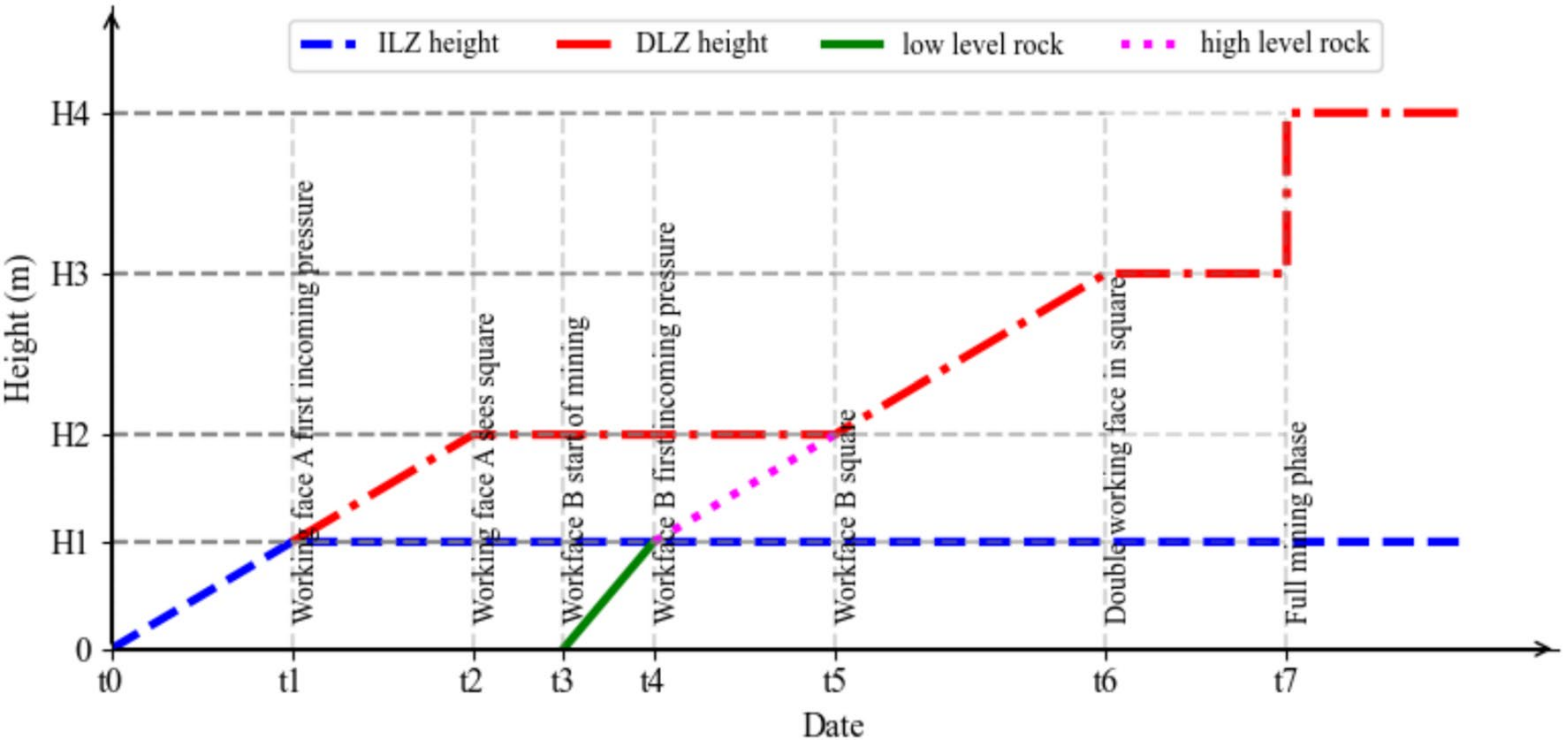
Simplified schematic diagram of the height evolution of the three load zones.

Based on the above analysis, a schematic diagram of the movement height of the strata in the Three Load Zones at different stages of mining can be drawn (Fig. 5).

In the first stage, as the recovery of working face A progresses, the immediate roof collapses, and the main roof sinks. The height of the overlying strata that undergoes movement gradually increases. The initial loading (t1) marks the point where the overlying strata movement height approaches the height range of the ‘ILZ’ zone. In the subsequent recovery stages, if mining conditions remain unchanged, the height H1 of the ‘ILZ’ zone remains relatively constant. Before the strata in the ‘ILZ’ zone move, the strata above it do not have space to move, so the thickness of the ‘DLZ’ zone is zero.

From t1 to t2, the collapse and compaction of the strata in the ‘ILZ’ zone create conditions for the movement of the strata in the overlying ‘DLZ’ zone. The fracture height of the ‘DLZ’ strata gradually increases from zero to H2, and the thickness of the ‘SLZ’ zone decreases.

The t2 stage marks the normal recovery stage of working face A, from the moment it begins to advance until the recovery is completed. During this stage, the thickness of the ‘DLZ’ zone remains unchanged. As mining progresses, the fracture position of the ‘DLZ’ strata above the goaf gradually advances upwards, but the maximum height does not exceed H2.

t3 marks the start of the recovery of working face B. Since the overlying strata of the adjacent goaf have already been damaged, the roof movement during the recovery of working face B is characterized by rapid and intense deformation. As a result, the height of the overlying strata destruction above working face B quickly reaches H1, with the first loading occurring at t4.

After the first loading, the height of the overlying strata destruction above working face B continues to increase, and it connects with the already-damaged roof of the adjacent working face, with the fracture height reaching H2.

After working face B has advanced, as mining continues, the minimum mining width of the mining area increases. The fracture height of the roof continues to rise, and the thickness of the ‘DLZ’ zone gradually increases, until both working faces advance to t6, with the fracture height reaching H3.

Once the mining area reaches fully mined conditions (here, t7 is considered a point in time for simplification), the maximum surface subsidence no longer increases with the expansion of the mining area. The thickness of the ‘SLZ’ zone reaches its minimum value, and the height of the ‘DLZ’ zone reaches its maximum value, H4.

### Differences between the three load zones and the traditional ‘three zones’

Unlike the traditional concept of the three zones (‘caving zone,’ ‘fracture zone,’ and ‘bending subsidence zone’), the division of the ‘Three Load Zones’ considers not only the spatial structure of the overlying strata but also the time effect of the stress exerted by the overlying strata on the underlying coal body. The primary goal is to control stress-induced disasters, rather than analyzing surface subsidence, water prevention, or gas control.

Firstly, the traditional three-zone concept divides the overlying strata based on the morphology of the rock layers after movement, while the ‘Three Load Zones’ model divides the zones based on the time-dependent stress impact of the strata movement on the underlying coal body. Therefore, the criteria for classification are different. The traditional three-zone concept focuses on the morphology of the strata, while the ‘Three Load Zones’ concept focuses on the stress loading method.

Secondly, since rock burst is a dynamic disaster, the division of the ‘Three Load Zones’ is significantly affected by time effects. The thicknesses of the ‘ILZ’ (Instantaneous Load Zone), ‘DLZ’ (Delayed Load Zone), and ‘SLZ’ (Static Load Zone) change with different mining stages and mining intensities. Under standard conditions, the ‘ILZ’ spatially includes the ‘caving zone’ and part of the faster-moving ‘fracture zone.’ The ‘DLZ’ spatially includes the slower-moving ‘fracture zone’ and the ‘bending subsidence zone,’ where rock fractures may occur. The ‘SLZ’ is the part of the ‘bending subsidence zone’ that can be regarded as uniformly distributed loading strata.

Thirdly, the establishment of the ‘Three Load Zones’ conceptual model aims to control dynamic disasters. It does not involve applications related to surface subsidence analysis, water prevention, or gas control.

Therefore, for the prevention and control of rock burst, the standard ‘ILZ’ + ’DLZ’ + ’SLZ’ three-zone structure is the key area of research. It should be noted that the calculation method for the thickness of the ‘Three Load Zones’ mentioned above is mainly applicable to general strata. When calculating the height of the ‘Three Load Zones,’ the estimation formula mentioned earlier is used as a first step. If the strata conditions are particularly distinctive (e.g., the presence of extremely thick and hard strata), the estimated results may deviate from actual values. In such cases, it is recommended to combine borehole column data and microseismic monitoring. The heights of the ‘Instantaneous Load Zone’ (ILZ) and ‘Delayed Load Zone’ (DLZ) can be determined by analyzing the height of microseismic event locations.

## Analysis of the evolutionary patterns of the ‘three load zones’ based on microseismic monitoring

### The characterization of the evolution of the three load zones based on microseismic indicators

The movement of the strata in the ‘ILZ’ and ‘DLZ’ zones of the Three Load Zones significantly affects the magnitude and distribution of the support stress in the underlying working face. The specific range is determined by the mining dimensions of the working face. Based on the variation in the minimum width of the goaf, the entire mining process is divided into the following stages, and the evolution of the Three Load Zones is analyzed accordingly.

**Fig. 6:**
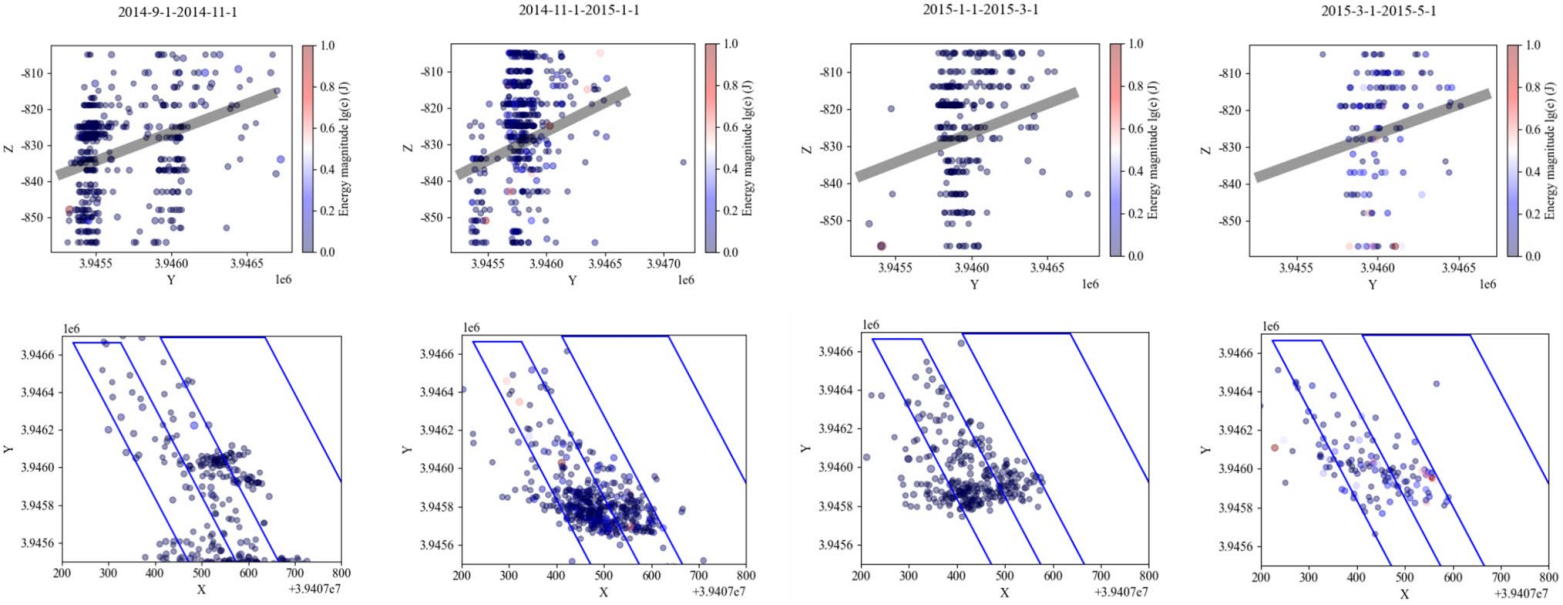
Incoming pressure at the working face revealed by microseismic monitoring.

**Fig. 7:**
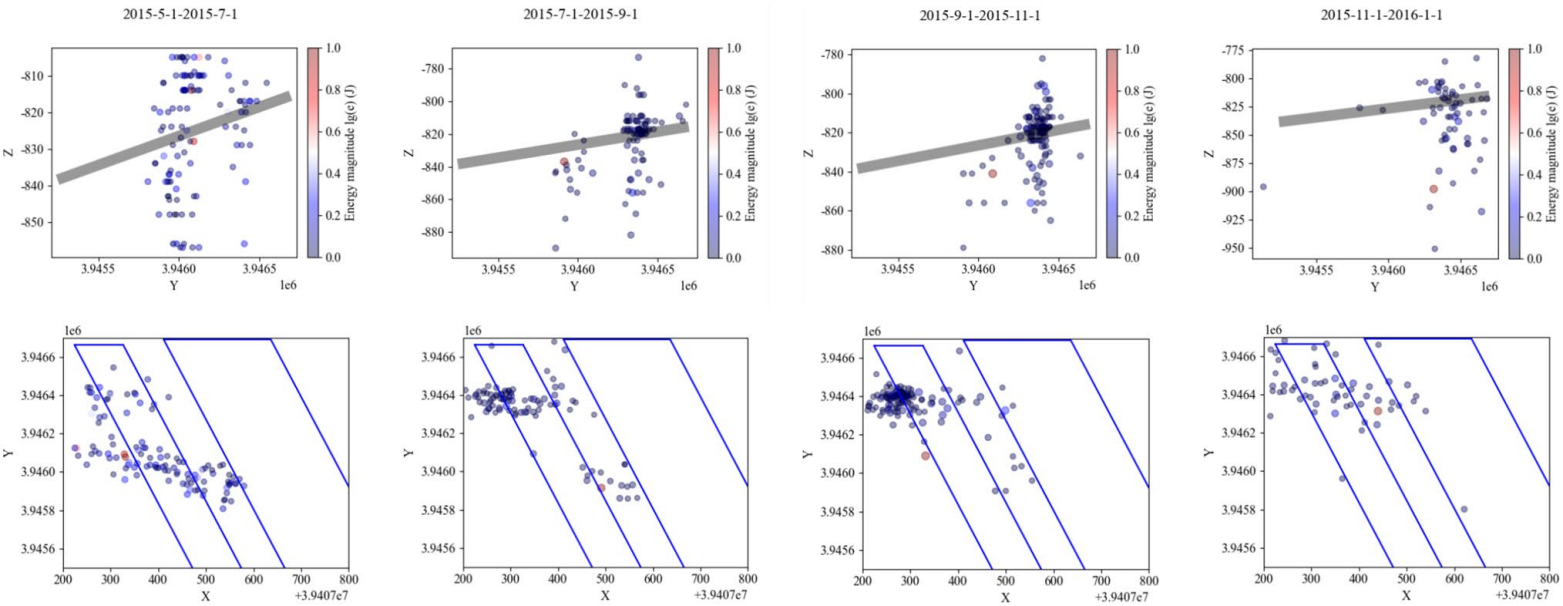
Working face square revealed by microseismic monitoring.

**Fig. 8:**
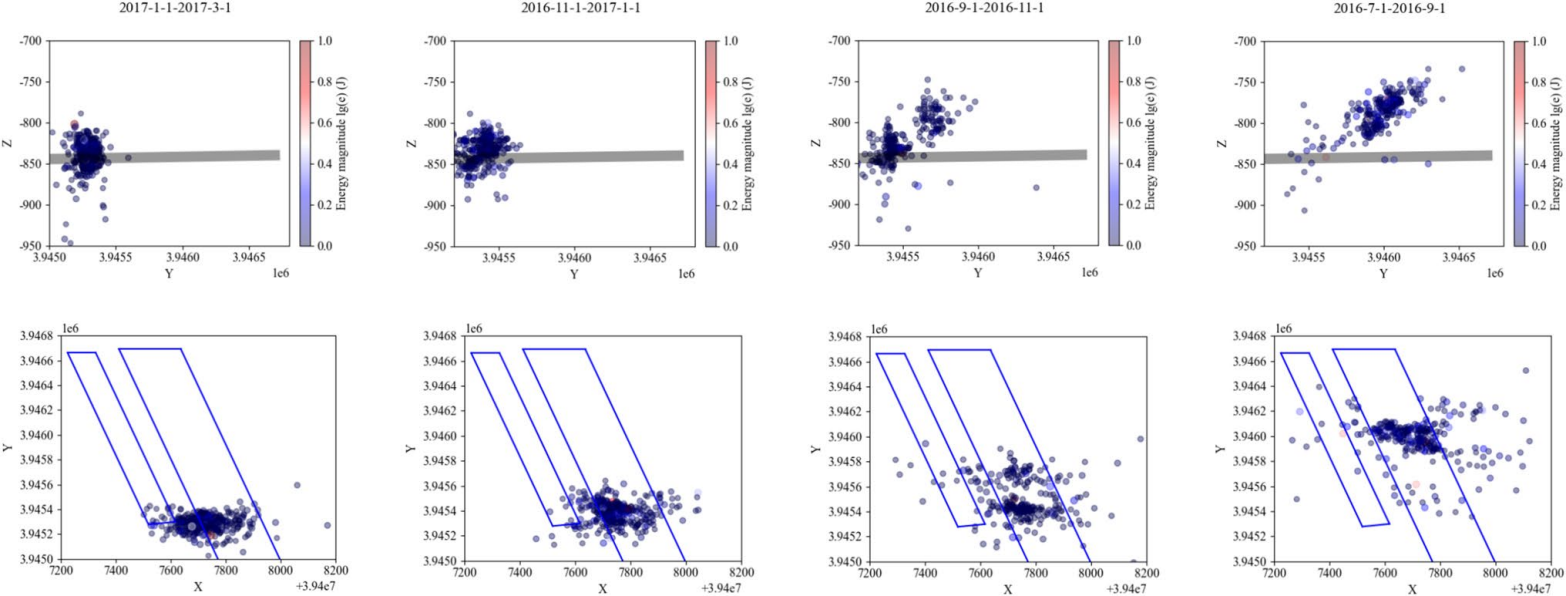
Double working surface square revealed by microseismic monitoring.

1. Formation Stage of the Preparation Roadway (First Stage): The side drifts and the cut-off for the first mining face A are formed. At this point, the roadway surrounding the working face is entirely coal. Due to the effect of support measures, there are no caving strata above the roadway, although small-scale roof delamination may occur.
2. Initial Loading Stage for Working Face A (Second Stage, see Fig. 6): Mining at working face A begins from the cut-off, and the goaf gradually expands. The immediate roof collapses and the main roof fractures. The occurrence of the initial loading marks the formation of the stress influence zone of the ‘ILZ’ zone in the mining area. The forward support pressure load from the roof movement begins to manifest. Since the minimum width of the goaf is still small at this stage, the ‘DLZ’ zone has not yet formed on a large scale, and there is no significant suspended roof. Therefore, the stress affecting the rock burst risk at this stage is mainly controlled by the forward support pressure in the strike direction of the ‘ILZ’ zone. The overlying strata structure is in a ‘ILZ’ + ’SLZ’ two-zone configuration.
3. Working Face A Advance Stage (Third Stage, see Fig. 7): After the initial loading, as the goaf gradually expands, working face A reaches the advancing position. At this point, the maximum height of the overlying strata that can form a structure reaches about half of the short side width of the goaf, and *H*_*DLZ*_ reaches its maximum value. The strata in the ‘DLZ’ zone, located above the ‘ILZ’ zone and with a thickness of *M*_*DLZ*_, will move slowly over a long period, gradually exerting stress on the underlying mining area. This results in a relatively large lateral support pressure and forward support pressure.
4. Initial Loading Stage of the Goaf Mining at Working Face B (Fourth Stage): After the completion of working face A, the stable roof structure of the goaf has already formed. As mining progresses at working face B, the previously stabilized roof structure is disrupted. The overlying strata above working face B, at the same height as the ‘ILZ’ zone of working face A, will undergo intense movement during the recovery process, and dynamic pressure within the range of forward support pressure will be significantly noticeable. Meanwhile, the fracture and connection of the roof at working face B with the roof of the goaf at working face A will lead to an increased range of forward support pressure on the goaf side. At this point, the stress primarily affecting the rock burst risk at working face B is the forward support pressure in the strike direction and the transmitted lateral stress.
5. Working Face B Advance Stage (Fifth Stage): During the mining of working face A, the strata within the height of the ‘DLZ’ zone have already undergone fracturing and subsidence. Therefore, during the mining of working face B, the movement of the ‘DLZ’ zone strata at this working face will accelerate significantly. When working face B reaches the advancing position, the ‘DLZ’ zone at this working face will quickly fracture to half the short side width of the goaf at this working face.
6. Dual Working Face Advance Stage (Sixth Stage, see Fig. 8): After working face B reaches the advancing position, the roof strata above the goaf at working face A, which had not fractured, also begin to destabilize, experiencing slow subsidence and fracturing. The strata originally within the ‘SLZ’ zone enter the range of the ‘DLZ’ zone. The thickness of the ‘SLZ’ zone decreases, and the thickness of the ‘DLZ’ zone increases until both working faces reach the advancing position. At this point, the thickness of the ‘DLZ’ zone strata reaches the maximum value for dual working face mining. As the thickness of the ‘DLZ’ zone strata increases, the strata involved in movement rise higher, and the stress influence range becomes larger. If there are hard, thick strata within the ‘DLZ’ zone, the working face will face the threat of ‘mine tremors’ (seismic events).
7. Full Mining Stage with Multiple Working Faces (Seventh Stage): With the continuous mining of multiple working faces in the mining area, a surface subsidence basin forms^31^. The stress influence range of the ‘Three Load Zones’ within the mining area no longer increases with the expansion of the mining area.

### Validation of the effectiveness of the ‘three load zones’ theory

To further validate the effectiveness of the ‘Three Load Zones’ theory, statistical analysis of high-precision microseismic monitoring data was conducted to reveal the movement patterns of the strata in the recovery working face mentioned earlier, and to compare and verify the validity of the ‘Three Load Zones’ theory.

**Fig. 9:**
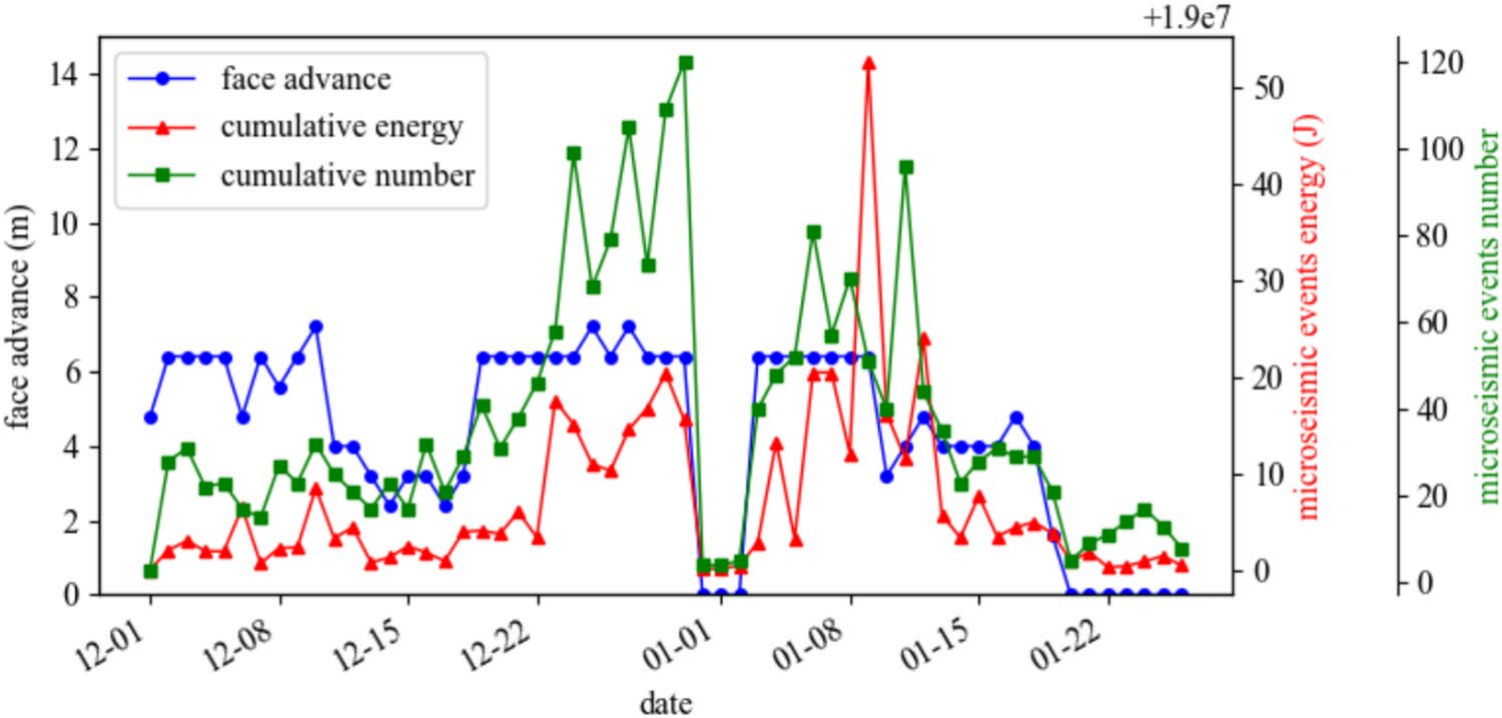
Figure of the number of microseismic events versus daily footfalls.

**Fig. 10:**
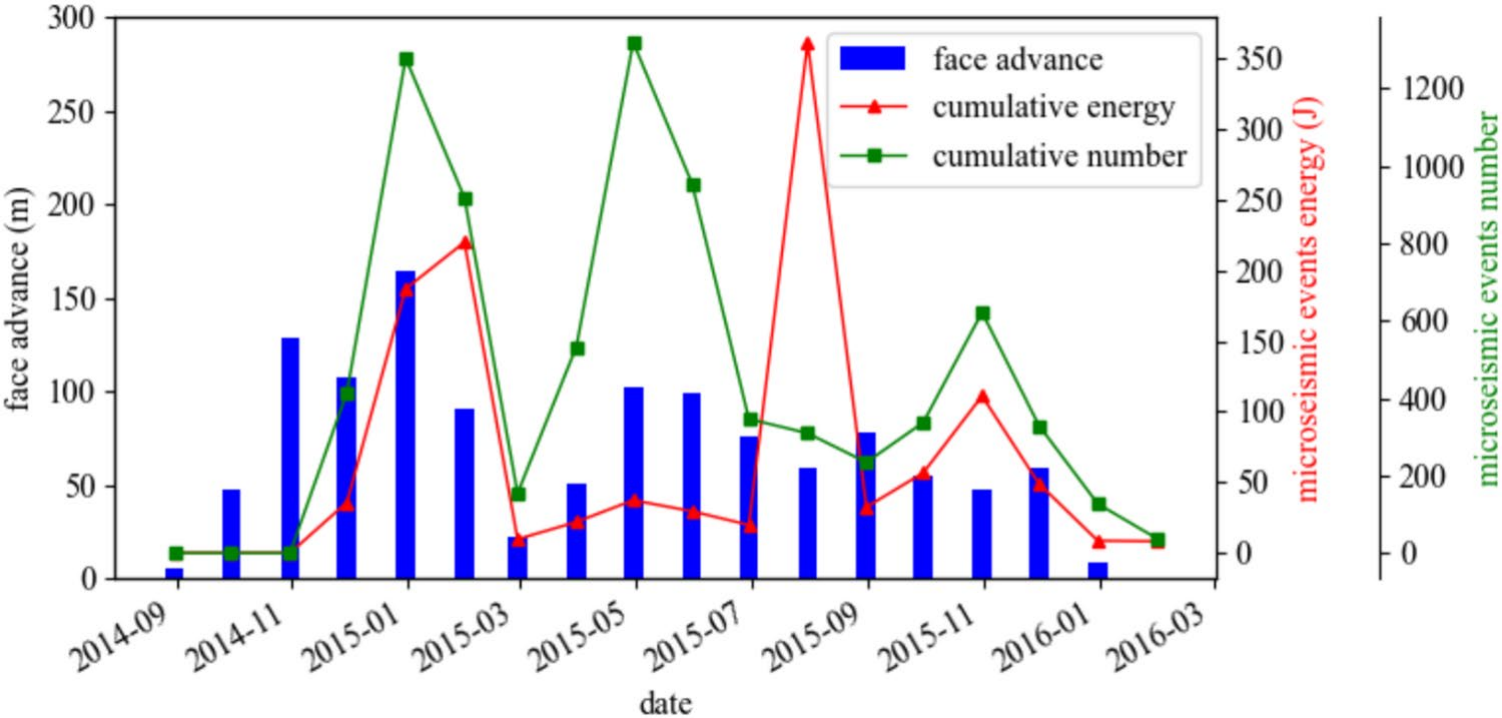
Statistical analysis of microseismic event count near the no. 2 crosscut area.

1. Relationship Between the Movement of Strata in the ‘ILZ’ Zone and Microseismic Events: Based on the results from the microseismic monitoring system at Yuncheng Coal Mine, a graph was plotted showing the relationship between the number of microseismic events and daily advance rate for the 1300 working face, as shown in Fig. 9. The graph shows a positive correlation between the number of microseismic events and the working face advance speed. The overlying ‘Instantaneous Load Zone’ strata, which move along with the advancing working face, fully reflect the characteristic of the ‘Instantaneous Load Zone’ undergoing periodic flexing, cracking, and collapse in the short term as mining progresses, thus transferring load to the surrounding coal and rock body of the working face.
2. Relationship Between the Movement of Strata in the ‘DLZ’ Zone and Microseismic Events: Multiple rock burst incidents occurred around the No. 2 crosscut area of the 1300 working face drainage roadway, and this area was designated as the key monitoring region. The number of microseismic events and the relationship between the working face advance distance and time were examined, and the data is plotted in Fig. 10.

From the graph, it can be seen that after the cut-off for the working face was formed in June, the number of microseismic events gradually decreased. As the working face began mining in August 2014, the number of microseismic events near the No. 2 crosscut significantly increased. As the working face gradually moved away from this area, microseismic events continued to increase in the short term and then gradually decreased. After the high-energy event on January 9, the number of microseismic events rapidly decreased, and subsequent mining showed a periodic variation pattern. This indicates that even after the working face moved away from the monitored area, the overlying strata in the region continued to move for a long period, transferring stress further into the surrounding coal and rock body of the goaf. This delayed movement and delayed loading characteristic aligns with the predictive conclusion of the ‘Delayed Load Zone’ model.

Due to the higher position of the ‘Delayed Load Zone,’ its lateral stress transfer influence range is larger. As the strata gradually move, the range of influence and the magnitude of the transferred stress will gradually increase, until the entire ‘Delayed Load Zone’ reaches a temporary stability. Therefore, the high-energy microseismic event recorded by the microseismic monitoring system on January 9 occurred in a coal pillar that was relatively far from the 1300 working face transport roadway (approximately 70 m in vertical distance). At the time of the event, the working face was also relatively far from this area (about 300 m).

As shown in Figs. 8, 9 and 10, the distribution of microseismic events exhibits clear regularity and zonation within the “Three-Zone Loading” model: (1) Vertical Distribution: 87.6% of microseismic events are concentrated in the ILZ (0–22 m) and DLZ (22–140 m) zones. Notably, all high-energy events (> 1 × 105 J) occur at key stratum rupture locations within the DLZ zone. (2) Temporal Correlation: 92% of microseismic events occur within ~ 2 h after periodic weighting in the ILZ zone. A sudden increase in microseismic frequency is observed 24 h before key stratum rupture in the DLZ zone. While the SLZ zone remains stable in the short term, microseismic activity—particularly high-energy events—gradually increases over time.

## Stress estimation model of the ‘three load zones’ and rock burst risk analysis

### Stress estimation model of the ‘three load zones’

Based on the ‘Three Load Zones,’ and combining the large amount of high-precision microseismic and stress field measurement data accumulated by the research team over many years from more than ten mines in Shanxi and Shandong, a theoretical model for static stress estimation was established through abstraction and summarization^17^. In the strike direction, the magnitude and distribution of the forward support pressure of the working face vary under three conditions of the ‘DLZ’ zone: complete suspended roof, partial suspended roof, and fully in contact with the goaf; stress estimation in the dip direction is primarily focused on serving the mining of adjacent working faces. Therefore, during analysis, it is assumed that the ‘DLZ’ zone has fully moved and is in a state of full contact with the goaf, which can be compared to the strike support pressure distribution under the same conditions. Based on the three stages of suspended roof in the ‘DLZ’ zone during the recovery process, three stress estimation models are established.

**Fig. 11:**
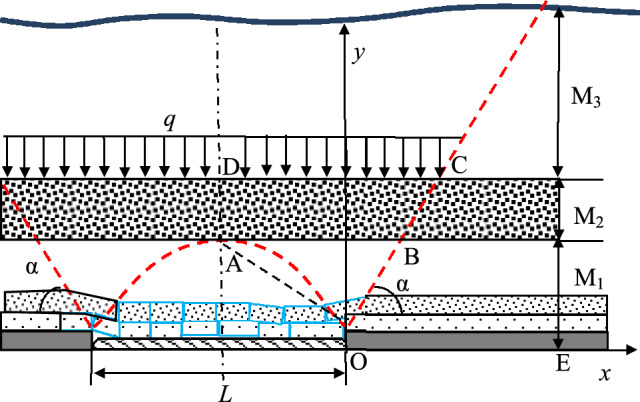
Stress estimate model of “DLZ” zone on wholly hanging condition.

(1) Complete Suspended Roof State of the ‘DLZ’ Zone: In deep mines with nearly horizontal mining areas, when the ‘DLZ’ zone is in the fully suspended roof stage, the estimation model for the forward support pressure of the mining face is shown in Fig. 11. The thicknesses of the ‘ILZ’ zone, ‘DLZ’ zone, and ‘SLZ’ zone are denoted as *M*_1_, *M*_2_, and *M*_3_, respectively. The line connecting the delamination point in front of the coal rib, denoted as OB, is called the strata movement boundary line. The angle between this line and the horizontal direction, denoted as *α*, is called the strata movement angle. *L* represents the minimum width of continuous mining in the mining area, and the dashed line AD represents the centerline of the goaf. An orthogonal coordinate system yox is established with the boundary point O between the goaf and coal rib as the origin, and the vertical stress on the coal body within the range of working face OE is calculated.

The vertical stress *σ* on the working face OE is composed of the self-weight stress *σ*_*y*_ of the overlying strata, the transmitted stress *σ*_*I*_ from the ‘ILZ’ zone strata structure, and the transmitted stress *σ*_*D*_ from the ‘DLZ’ zone strata structure. For the self-weight stress *σ*_*y*_, due to the stress transfer effect from the ‘DLZ’ zone above, the self-weight stress exerted by the overlying strata on working face OE differs significantly between areas close to the goaf and those further away.

The transmitted stress *σ*_*I*_ from the ‘ILZ’ zone originates approximately from the weight *Q*_*OAB*_ of the strata within the ‘ILZ’ zone in the OAB range, which forms a ‘structure.’ The mining method and the quality of the strata in the ‘ILZ’ zone will result in different locations for the goaf of this ‘structure,’ so the weight of the strata in this area may be partially or fully transferred to the coal body at working face OE. The distribution of the support pressure in front of the coal rib at the working face is determined by multiple factors. After approximating the measured results within the permissible engineering accuracy, a triangular distribution model is used.

4 $$\sigma_{I} = \left\{ \begin{gathered} \frac{{2\sigma_{{{\text{Im}} ax}} \tan \alpha }}{{M_{1} }}x,\begin{array}{*{20}c} {} & {} \\ \end{array} \left[ {0,\frac{{M_{1} }}{2\tan \alpha }} \right] \hfill \\ 2\sigma_{{{\text{Im}} ax}} \left( {1 - \frac{x\tan \alpha }{{M_{1} }}} \right),\left[ {\frac{{M_{1} }}{2\tan \alpha },\frac{{M_{1} }}{\tan \alpha }} \right] \hfill \\ 0,\begin{array}{*{20}c} {} & {} \\ \end{array} \left( {\frac{{M_{1} }}{\tan \alpha }, + \infty } \right) \hfill \\ \end{gathered} \right.$$

The value of the peak transmitted stress *σ*_*Imax*_ in the ‘ILZ’ zone is determined by the following equation:

5 $$\sigma_{{{\text{I}} ,\max }} = \frac{{Q_{OAB} K_{I} \tan \alpha }}{{M_{1} }} = \left( {\frac{L\tan \alpha }{4} + \frac{{M_{1} }}{2}} \right)\gamma K_{I}$$

In the equation, *L* represents the minimum span of continuous mining in the mining area; *K*_*I*_ is the transmitted stress coefficient of the ‘ILZ’ zone, which is related to the strata quality and mining advance speed of the ‘ILZ’ zone.

As shown in Fig. 10, the ‘DLZ’ zone above the ‘ILZ’ zone forms a ‘double-beam’ structure, with the ‘SLZ’ zone and its own self-weight stress being transmitted to the coal and rock body on both sides of the rock beam. For the working face OE, the transmitted stress *σ*_*D*_ is half of the total transmitted stress of the ‘DLZ’ zone:

6 $$\sigma_{D} = \left\{ \begin{gathered} \frac{{\sigma_{D\max } \tan \alpha }}{{M_{1} + \frac{{M_{2} }}{2}}}x,\begin{array}{*{20}c} {} & {} \\ \end{array} \left[ {0,\frac{{2M_{1} + M_{2} }}{2\tan \alpha }} \right] \hfill \\ \sigma_{D\max } \left( {2 - \frac{x\tan \alpha }{{M_{1} + \frac{{M_{2} }}{2}}}} \right),\left[ {\frac{{2M_{1} + M_{2} }}{2\tan \alpha },\frac{{2M_{1} + M_{2} }}{\tan \alpha }} \right] \hfill \\ 0,\begin{array}{*{20}c} {} & {} \\ \end{array} \left( {\frac{{2M_{1} + M_{2} }}{\tan \alpha }, + \infty } \right) \hfill \\ \end{gathered} \right.$$

The value of the peak transmitted stress *σ*_*Dmax*_ in the ‘DLZ’ zone is determined by the following equation:

7 $$\sigma_{D\max } = \frac{{\left( {Q_{ABCD} + Q_{{M_{3} }} } \right)\tan \alpha }}{{2M_{1} + M_{2} }} = \frac{{\left[ {\left( {\frac{L}{2} + \frac{{2M_{1} + M_{2} }}{2\tan \alpha }} \right)M_{2} + \left( {\frac{L}{2} + \frac{{M_{1} + M_{2} }}{\tan \alpha }} \right)M_{3} } \right]\gamma }}{{\left( {2M_{1} + M_{2} } \right)\cot \alpha }}$$

**Fig.12:**
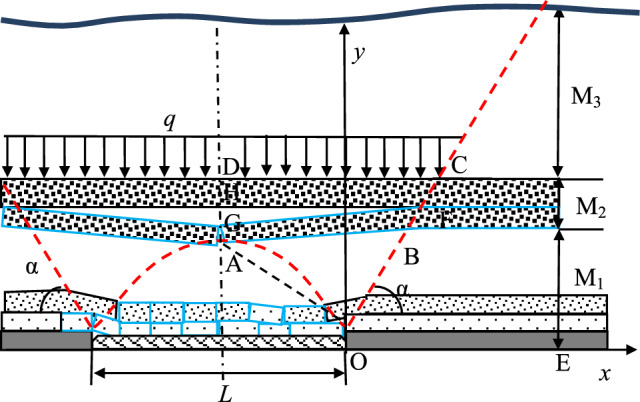
Stress estimate model of “DLZ” zone on partial hanging condition.

(2) Partial Suspended Roof State of the ‘DLZ’ Zone: As the ore body is mined, the strata in the ‘ILZ’ zone experience collapse and rotational subsidence within a short period, forming a structure. The shape of this structure does not change significantly over time. Therefore, in this stage, the transmitted stress *σ*_*I*_ in the ‘ILZ’ zone remains the same in magnitude and distribution as in the previous stage. After the ‘DLZ’ zone experiences a fully suspended roof state, the lower strata fracture and come into contact with the goaf due to the load they bear exceeding their strength, while the upper strata have not yet fractured, forming a partial suspended roof state, as shown in Fig. 12.

As shown in Fig. 11, the strata in the ‘DLZ’ zone, forming a structure above the ‘ILZ’ zone, on one hand, transmit the self-weight of the overlying ‘SLZ’ zone, and on the other hand, part of the self-weight is transmitted to the floor due to contact with the goaf. For the working face OE, the transmitted stress *σ*_*D*_ is half of the self-weight of the strata in the ABFG area of the ‘DLZ’ zone, as well as the total transmitted stress from the strata in the HFCD area. The distribution pattern is the same as in the previous stage, and the peak value *σ*_*Dmax*_ is calculated as follows:

8 $$\begin{gathered} \sigma_{D\max } = \frac{{\left( {\frac{{Q_{{AB{\text{FG}}}} }}{2} + Q_{HFCD} + Q_{{M_{3} }} } \right)\tan \alpha }}{{M_{1} + \frac{{M_{2} }}{2}}} \hfill \\ \begin{array}{*{20}c} {} & {} \\ \end{array} = \frac{{\left[ {\left( {\frac{L}{2} + \frac{{2M_{1} + M_{2} }}{2\tan \alpha }} \right)\gamma M_{2} + \left( {\frac{L}{4} + \frac{{2M_{1} + M_{D} }}{4\tan \alpha }} \right)\gamma M_{D} } \right]}}{{\left( {M_{1} + \frac{{M_{2} }}{2}} \right)\cot \alpha }} + \frac{{\left( {\frac{L}{2} + \frac{{M_{1} + M_{2} }}{\tan \alpha }} \right)\gamma M_{3} }}{{\left( {M_{1} + \frac{{M_{2} }}{2}} \right)\cot \alpha }} \hfill \\ \end{gathered}$$

In the equation, *M*_*D*_ represents the thickness of the fractured strata in the ‘DLZ’ zone, and its value is related to the suspension time and the strata quality of the ‘DLZ’ zone.

**Fig. 13:**
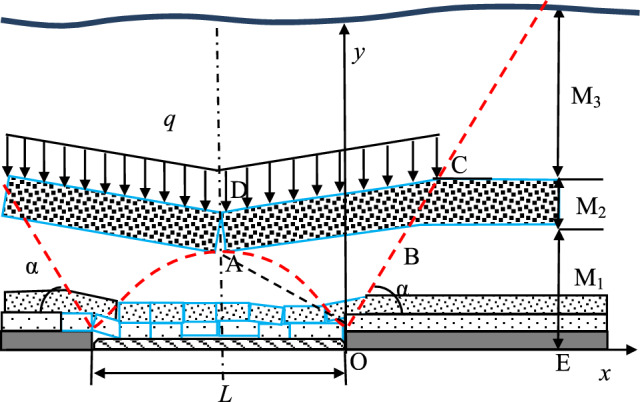
Stress estimate model of “DLZ” zone on fully sedimentary condition.

(3) Complete Contact with Goaf State of the ‘DLZ’ Zone: Under the determined mining width *L*, after the ‘DLZ’ zone has fully contacted the goaf, the strata below the rock beam are supported by the goaf refuse, while the strata above are subjected to the self-weight stress of the ‘SLZ’ zone. The transmitted stress to the coal body in front is approximately equal to the self-weight of the ‘SLZ’ zone in the DC section and half of the self-weight of the strata in the ABCD section. In this stage, the magnitude and distribution of the transmitted stress *σ*_*I*_ in the ‘ILZ’ zone remain the same as in the previous stage. The stress estimation model is shown in Fig. 13.

Since the overall height of the ‘DLZ’ zone remains unchanged, it is assumed that its distribution pattern remains constant. The value of the peak transmitted stress *σ*_*Dmax*_ is determined by the following equation:

9 $$\sigma_{D\max } = \frac{{\left( {\frac{{Q_{ABCD} }}{2} + Q_{{M_{3} }} } \right)\tan \alpha }}{{M_{1} + \frac{{M_{2} }}{2}}} = \frac{{\left( {\frac{L}{4} + \frac{{2M_{1} + M_{2} }}{4\tan \alpha }} \right)\gamma M_{2} + \left( {\frac{L}{2} + \frac{{M_{1} + M_{2} }}{\tan \alpha }} \right)\gamma M_{3} }}{{\left( {M_{1} + \frac{{M_{2} }}{2}} \right)\cot \alpha }}$$

When the mining area reaches the fully mined stage, the minimum mining width L and the height of the ‘DLZ’ zone no longer satisfy the dimensional relationship defined earlier, and therefore Eq. (9) no longer holds. However, during the fully mined stage, the strata in the ‘DLZ’ zone still satisfy the spatial model shown in Fig. 7.

### Stress analysis of rock burst caused by the movement of the three zones

According to the static stress estimation formula for the Three Load Zones, it can be seen that when the thickness of the Three Load Zones remains unchanged, the range of support pressure and the maximum static stress applied by the Three Load Zones to the coal body in the strike and dip directions are fixed, thus determining the base stress state of the coal and rock body. The movement of the ‘ILZ’ and ‘DLZ’ zones is the source of dynamic pressure that causes the rock burst.

#### Impact of changes in the ‘ILZ’ zone on rock burst risk

(1) Rock burst Risk Caused by Changes in the Thickness of the ‘ILZ’ Zone: In practical engineering, the mining height of the working face changes due to the influence of geological and mining conditions in the mining area. When factors such as increased coal thickness or roof leakage cause the mining height to increase, the thickness of the ‘ILZ’ zone will also increase. In this case: The distribution range of transmitted stress in the ‘ILZ’ zone expands. The height of the ‘ILZ’ zone changes from M1 to M1 + ΔM. According to Eq. (3), the increment in the transmitted stress distribution range ΔSI for the ‘ILZ’ zone is:

10 $$\Delta S_{I} = \frac{\Delta M}{{\tan \alpha }}$$

The peak transmitted stress of the ‘ILZ’ zone increases, and its location shifts. The increment in the peak stress *σ*_*Imax*_ is:

11 $$\Delta \sigma_{{{\text{Im}} ax}} = \Delta M\gamma K_{I} \left( {\frac{L\tan \alpha + 2\Delta M}{{4M_{1} }} + 1} \right)$$

The stress increment Δ*σ*_*Imax*_ is one of the key factors influencing the rock burst risk due to the mining height of the working face.

(2) Impact of Mining Advance Speed on Rock burst Risk: An increase in the mining advance speed will lead to a longer fracture cycle of the ‘ILZ’ zone, causing the contact line to move further away from the coal rib and increasing the volume of suspended strata, which in turn results in an increase in the support pressure transmitted to the coal body in the OE range.

(3) Impact of the Strata Quality in the ‘ILZ’ Zone on Rock burst Risk: An increase in the quality of the strata in the ‘ILZ’ zone means that the roof is less prone to fracture, leading to an increased distance to the suspended roof and a longer cycle of loading. This also causes the contact line to move further away from the coal rib, thereby increasing the support pressure and raising the rock burst risk.

#### Impact of changes in the ‘DLZ’ zone on rock burst risk

(1) Impact of Changes in the Thickness of the ‘DLZ’ Zone on Rock burst Risk: As the mining area expands, before reaching fully mined conditions, the height of the ‘DLZ’ zone will increase with the enlargement of the minimum span of the goaf. According to Eqs. (8) and (9), it can be seen that during the recovery process of the working face, two changes will occur in the transmitted stress of the ‘DLZ’ zone:

The distribution range of the transmitted stress in the ‘DLZ’ zone expands. Its specific numerical calculation model is analogous to the ‘ILZ’ zone, and will not be recalculated here. The expansion of the transmitted stress range will increase the extent of the rock burst risk area.

The peak transmitted stress of the ‘DLZ’ zone changes. According to the definition of the Three Load Zones, the sum of the thicknesses of the Three Load Zones is equal to the mining depth H. The increase in M2 will cause M3 to decrease. Based on Eq. (9), the increase or decrease in the peak stress needs to be determined according to specific on-site conditions. As the range of the forward support pressure expands, the location of the peak stress will shift to deeper coal body regions.

(2) Impact of State Transitions in the ‘DLZ’ Zone on Rock burst Risk: During the transition from the fully suspended roof state to the fully in contact with the goaf state in the ‘DLZ’ zone, the strata at higher levels will fracture. If the strata in the ‘DLZ’ zone have good quality, the fracture will release a large amount of energy instantaneously, leading to a ‘mine earthquake’ disaster, and possibly triggering a rock burst induced by the mine earthquake. The transition time between the three states of the ‘DLZ’ zone is determined by the quality of the strata. If the strata quality is good, the suspended roof time will be longer, and the mining roadway will continuously be affected by the transferred stress for an extended period.

## Field validation and application of the ‘three load zones’ model

**Fig. 14:**
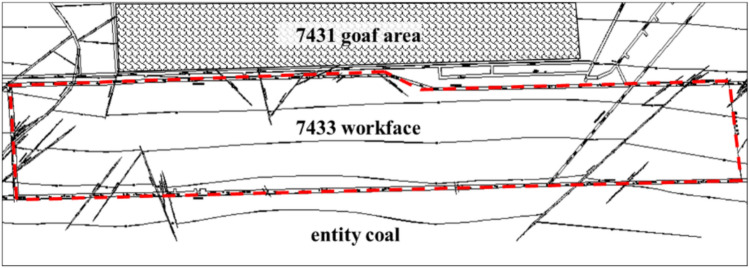
7433 working face engineering plan.

To validate the effectiveness of the above method, a comparative study was conducted using Kongzhuang Coal Mine as an example, based on the ‘Three Load Zones’ theory, microseismic field measurements, and numerical simulation analysis. The average mining depth of the 7433 goaf mining face at Kongzhuang Coal Mine is 900 m, with an inclined length of approximately 130 m and a strike length of 1246 m. The coal seam thickness is 4.6 m, with a simple structure, generally a monocline with an average dip angle of 21°, and numerous faults in the area. The uniaxial compressive strength of the coal seam is 20 MPa, indicating a rock burst tendency. The immediate roof is sandstone (6.66 m thick), and the main roof consists of sandstone, mudstone, and fine-grained sandstone (17.1 m thick). The working face is mined along the goaf and is adjacent to the 7431 goaf area. The dip width is 130 m, and the coal pillar width is 6 m, as shown in Fig. 14.

### Structural analysis of the three load zones in the working face

According to the definition of the Three Load Zones and based on the mining dimensions of the 7433 working face, the height of the Three Load Zones is determined. From the stratigraphic column, it can be seen that the immediate roof of the 7433 working face is sandstone, and the main roof consists of a layer group of sandstone, mudstone, and fine-grained sandstone. In the strata above the coal seam, extending to the surface, there are multiple layers of hard medium-grained sandstone and sandstone-mudstone interbedded structures. The coal seam thickness in the 7433 working face is approximately 4.6 m, and top-coal caving mining is used. Based on an 80% mining recovery rate, the mined height is 3.68 m. The 7433 working face is mined along the goaf. As shown in Fig. 16, it can be seen that the roof fracture height above the roadway on the 7431 goaf side is larger, while the roof fracture height on the solid coal side is smaller. From the solid coal side to the goaf side, the roof fracture height gradually increases, forming an S-shaped overlying strata spatial structure. Due to the different roof fracture heights in the two roadways, the Three Load Zones structure and dimensions also differ, and need to be analyzed separately. As shown in Fig. 17, cross-sections A-A and B-B are made perpendicular to the roof fracture line. Since the angle between the coal seam floor and the horizontal plane in both sections is relatively small (about 15°), it can be approximated as horizontal. The fracture angle *α* of the strata in the cross-sectional direction is taken as the average of the two roadways, which is 55°. According to the microseismic positioning results, it can be seen that in the A-A cross-section, the strata above the ‘DLZ’ zone at the belt roadway position are either just fractured or not fractured yet. In the B-B cross-section, at the track roadway position, the strata above the ‘DLZ’ zone are already mostly in contact with the goaf. Therefore, the dimensions and shape of the ‘DLZ’ zone are the main factors determining the stress state of the two roadways.

(1) Three Load Zones Structure and Dimensions at the A-A Cross-Section: Microseismic monitoring results show that the fracture height of the strata at the belt roadway position in the A-A cross-section is approximately 70 m. According to the Three Load Zones theoretical model, this position is in a fully suspended roof state for the ‘DLZ’ zone.

- ‘ILZ’ zone thickness M1: M1 ≈ 10h = 36.8m
- ‘DLZ’ zone thickness M2: M2 = (70—36.8)m = 33.2 m
- ‘SLZ’ zone thickness M3: M3 = (900—70)m = 830 m

(2) Three Load Zones Structure and Dimensions at the B-B Cross-Section: Microseismic monitoring results show that the fracture height of the strata at the track roadway position in the B-B cross-section is approximately 130 m. According to the Three Load Zones theoretical model, this position is in the contact with the goaf state for the ‘DLZ’ zone.

- ‘ILZ’ zone thickness M1: M1 ≈ 10h = 36.8m
- ‘DLZ’ zone thickness M2: M2 = (130—36.8)m = 93.2 m
- ‘SLZ’ zone thickness M3: M3 = (900—130)m = 770 m

### Estimation of forward support pressure and its influence range

Based on the Three Load Zones structure in the A-A and B-B cross-sections, forward support pressure estimation models are established. The thicknesses of the ‘ILZ’ zone, ‘DLZ’ zone, and ‘SLZ’ zone are denoted as *M*_1_, *M*_2_, and *M*_3_, respectively. The line connecting the delamination point in front of the coal rib, denoted as OB, is called the strata movement boundary line. The angle between this line and the horizontal direction, denoted as *α*, is called the strata movement angle. *L* represents the width of the goaf, and AD represents the centerline of the goaf. An orthogonal coordinate system yox is established with the boundary point O between the goaf and coal rib as the origin, and the vertical stress on the coal body within the range of OE is calculated.

The vertical stress σ on solid coal OE is composed of the self-weight stress *σ*_*y*_ of the overlying strata, the transmitted stress *σ*_*I*_ from the ‘ILZ’ zone strata, and the transmitted stress *σ*_*D*_ from the ‘DLZ’ zone strata, i.e., *σ* = *σ*_*y*_ + *σ*_*I*_ + *σ*_*D*_.

**Fig.15:**
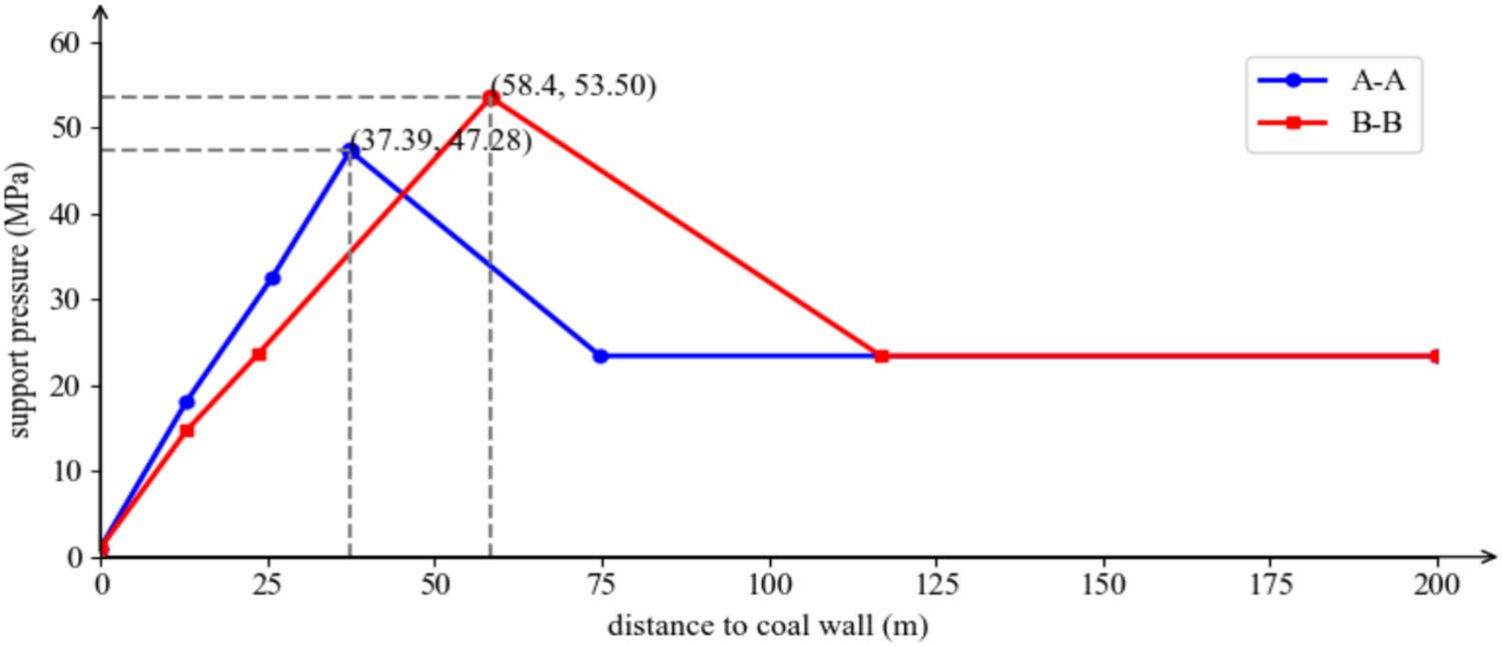
A-A and B-B cross-section forward support pressure curves.

(1) Stress Estimation at the Belt Roadway Position in the A-A Cross-Section: When the ‘DLZ’ zone in a deep mine with nearly horizontal mining is in the fully suspended roof stage, the forward support pressure estimation model for the mining area is shown in Fig. 11. Based on the static stress estimation formula previously defined, the stress distributions of *σ*_*y*_, *σ*_*I*_, and *σ*_*D*_ are calculated separately. By superimposing them, the forward support pressure curve in the A-A cross-section, shown in Fig. 15, is obtained.

From the figure, it can be seen that the peak forward support pressure is 47.28 MPa, and the maximum influence range is 74.78 m.

(2) Stress Estimation at the Track Roadway Position in the B-B Cross-Section: When the ‘DLZ’ zone in a deep mine with nearly horizontal mining is in the contact with the goaf state, the forward support pressure estimation model for the mining area is shown in Fig. 13. Similarly, the stress distributions of *σ*_*y*_, *σ*_*I*_, and *σ*_*D*_ are calculated separately. By superimposing them, the forward support pressure curve in the B-B cross-section, shown in Fig. 15, is obtained. From Fig. 15, it can be seen that the peak forward support pressure is 53.50 MPa, and the maximum influence range is 116.79 m.

### Comparative analysis and validation of other methods

#### Analysis of microseismic monitoring results

To monitor the movement of the overlying strata in the 7433 goaf mining face and provide rock burst early warning, the mine installed a microseismic monitoring system. The seismic detectors are arranged within the influence range of the working face mining activities, allowing real-time monitoring of rock body fracturing events during the recovery process. Based on the microseismic positioning results, the movement patterns of the strata can be inferred, enabling timely understanding of the roof condition and providing early warning for rock burst.

**Fig. 16:**
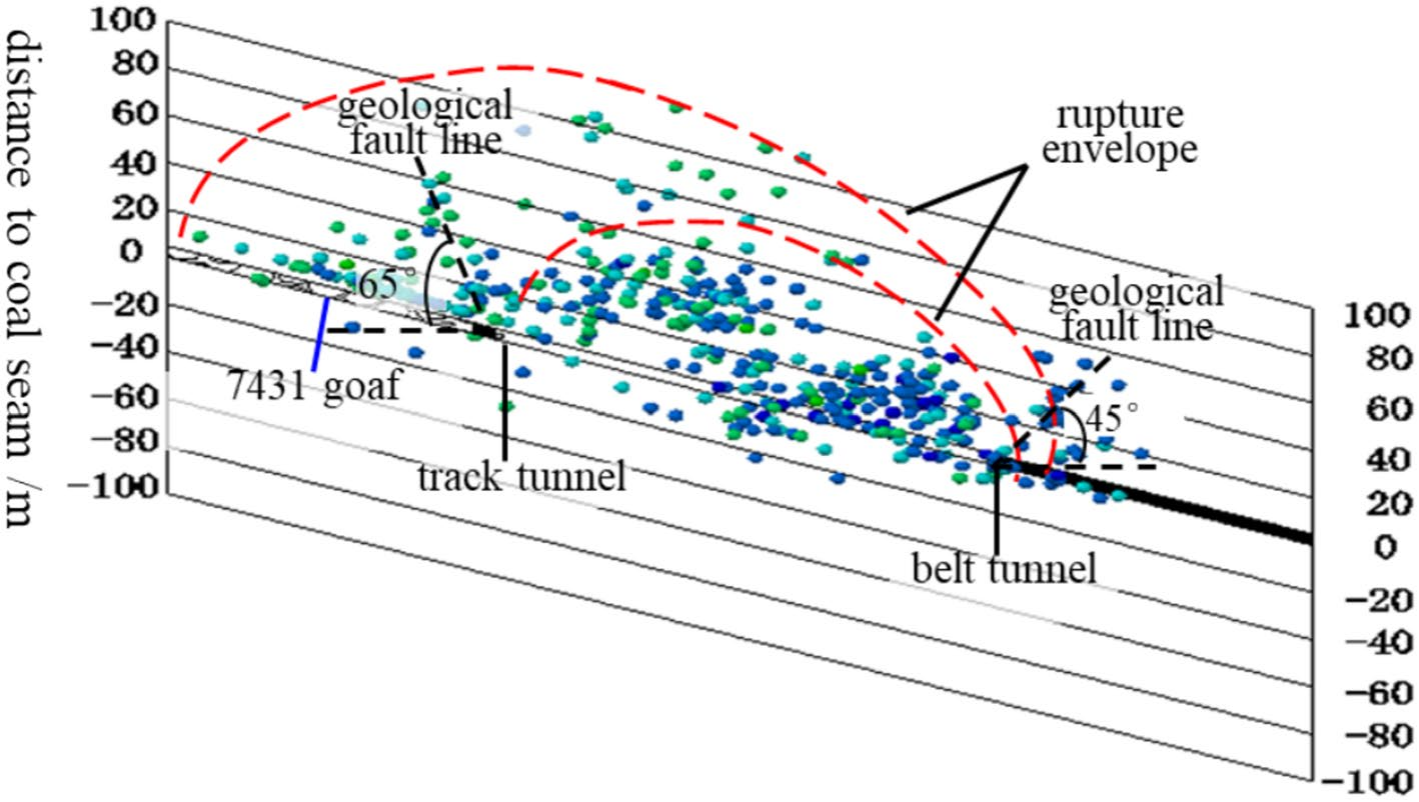
Rupture map of quarry rock layers revealed by microseismic monitoring.

**Fig. 17:**
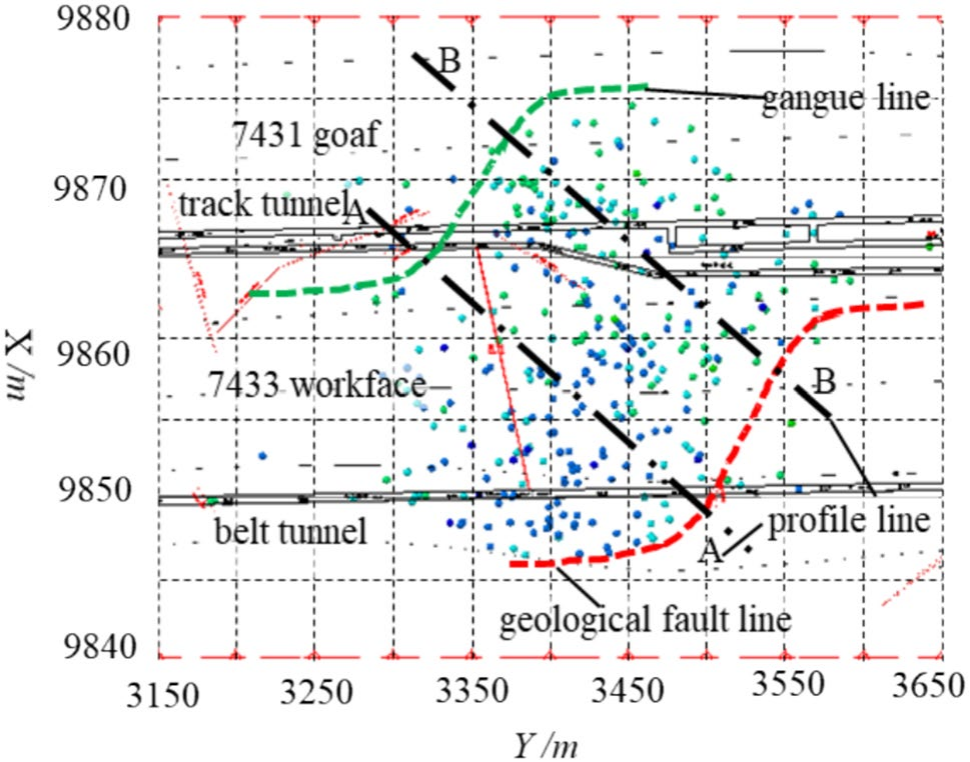
7433 fixed working face strike plan projection.

**Fig. 18:**
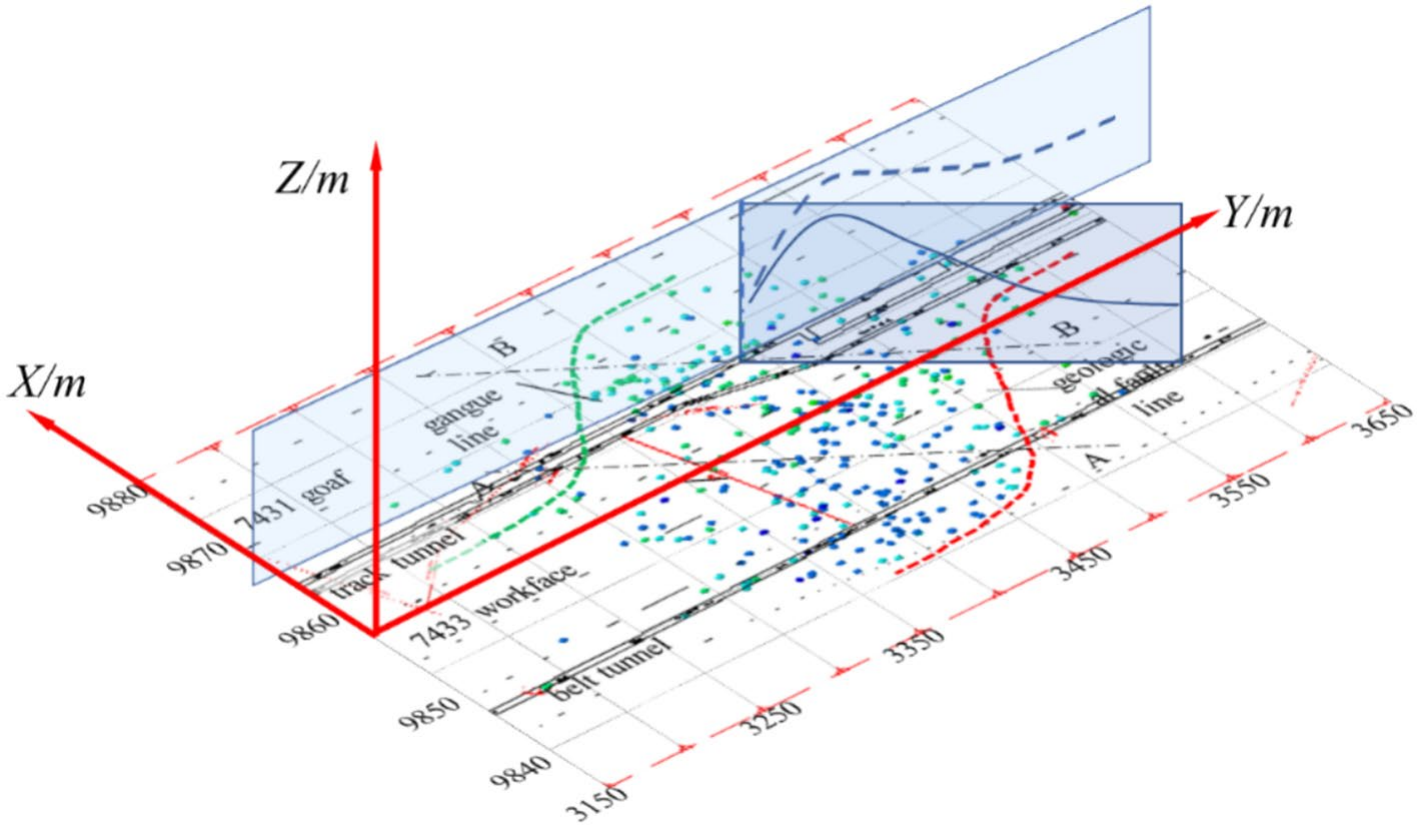
Schematic projection of the stress influence range.

**Fig. 19:**
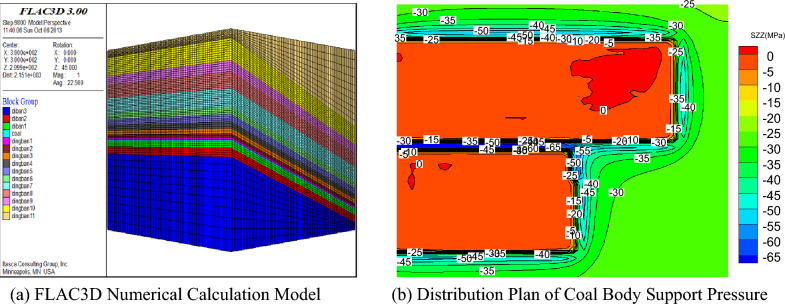
FLAC3D numerical calculation model and result analysis.

This study analyzed and visualized microseismic data using Python for statistical processing and AutoCAD scripting for 3D parametric modeling (Figs. 16, 17, 18), projecting seismic events onto the Y–Z plane while deriving stratum boundaries from geophysical data. Numerical simulations in FLAC3D (Fig. 19) computed abutment pressure distributions, visualized as contour maps using Tecplot 2022 R1, integrating computational geomechanics with advanced data visualization techniques.

Based on the microseismic monitoring results, the extent and range of surrounding rock damage in the working face can be determined. We performed statistical analysis and visualization of microseismic data using Python. Figure 16 shows the development of the fracture field along the height direction of the working face, as revealed by microseismic monitoring. From the projection of the microseismic events, it can be seen that the fracture height of the strata on the track roadway side of the 7433 working face is approximately 130 m, while the fracture height of the strata in the goaf is about 70 m.

Based on the microseismic positioning results, the fracture lines of the strata on the track roadway side and the belt roadway side were plotted. It can be measured that the angle between the fracture line of the strata on the track roadway side and the horizontal plane is approximately 65°, while the angle between the fracture line of the strata on the belt roadway side and the horizontal plane is approximately 45°. Figure 17 shows the fixed working face projection of microseismic events in the 7433 working face. In the figure, the X-axis represents the dip direction of the working face, and the Y-axis represents the strike direction of the working face. The track roadway side is the 7431 goaf, and the belt roadway side is the solid coal. From Fig. 17, it can be seen that the microseismic events are concentrated between the contact line and fracture line shown in the figure, and the mining area exhibits a typical S-shaped overlying strata spatial structure. The forward influence range on the goaf side of the working face is about 210 m, while the forward influence range on the solid coal side of the working face is about 100 m.

From Fig. 16, it can be seen that the A-A and B-B cross-sections form an angle of approximately 50° with the roadway. Therefore, to determine the forward support pressure influence range in the double-sided roadways, the stress influence range in the cross-sectional direction needs to be projected onto the roadway direction based on the angle between the cross-sectional line and the roadway, as shown in Fig. 18. In the figure, the X-axis is parallel to the dip direction of the working face, the Y-axis is parallel to the strike direction, and the Z-axis represents the vertical direction. The origin of the coordinate system is located at the center point of the open-off cut.

Using the method illustrated in the figure, the stress influence ranges of the A-A and B-B cross-sections are projected separately. The results are as follows: On the belt roadway side, the forward support pressure influence range is approximately 116.34 m; on the track roadway side, the forward support pressure influence range is approximately 181.70 m. The theoretical calculation results are generally consistent with the results obtained from microseismic monitoring, though some discrepancies exist. To verify and visually demonstrate the distribution of the forward support stress field, numerical simulation methods are used for further analysis.

#### Validation of numerical simulation analysis results

A numerical model was established based on the stratigraphic column and mining conditions of the 7433 working face and simulated using FLAC3D. Figure 19 presents the simulation results, generated as follows: (a) the geometric model was constructed in FLAC3D; (b) the abutment pressure distribution was computed through FLAC3D simulation and visualized as a contour map. The numerical calculation model includes 15 strata groups, as shown in Fig. 19a. The boundary conditions of the model are horizontal displacement constraints on all sides, vertical displacement constraints at the bottom, and a uniformly distributed load at the top. In the vertical direction, stress increases linearly with depth, while in the horizontal direction, the stress is taken as half of the vertical stress. Figure 19b shows the distribution plan of the working face support pressure after the working face has advanced 300 m and entered the stable mining stage.

From the figure, it can be seen that on the goaf side of the coal body, the peak forward support pressure of the working face is approximately 55 MPa, with a forward influence range of about 200 m. On the solid coal side, the maximum forward support pressure of the working face is 45 MPa, with an influence range of about 80 m.

### Comparative analysis of results from three methods

A comparative analysis of the results from the Three Load Zones theory derivation, microseismic monitoring, and numerical simulation methods is conducted, and the results are shown in Table 2.

**Table 2:** Comparison of numerical simulation, microseismic monitoring and theoretical calculation results.

Methodology	Peak advance abutment pressure /MPa		Advance influence scope /m	
	Belt tunnel	Truck tunnel	Belt tunnel	Truck tunnel
FLAC^3D^ numerical simulation	45	55	80	200
Theoretical calculation	47.28	53.50	116.34	181.70
Microseismic monitoring	–	–	100	210

As shown in the table above, the results obtained from the three research methods are generally consistent. Both the numerical simulation and theoretical calculation results indicate that the support pressure level in the roadway on the goaf side (track roadway) is relatively high. The uniaxial compressive strength of the mined coal seam is 20 MPa, and the ratio of the maximum stress to the coal body’s uniaxial compressive strength reaches 2.75 > 1.5. According to the macro criteria for rock burst, if effective rock burst prevention and support measures are not implemented in the goaf side roadway, the track roadway of the 7433 working face is highly likely to experience rock burst. It can be concluded that the conclusions drawn from the ‘Three Load Zones’ theory proposed in this paper are accurate and practical.

## Discussion and conclusion

### Discussion

The ‘Three Load Zones’ strata structure model is an important innovation in the study of rock burst stress fields. This model divides the mining area into three parts: the Static Load Zone (SLZ), the Delayed Load Zone (DLZ), and the Instantaneous Load Zone (ILZ). By analyzing the structural characteristics and movement patterns of the different load zones, the model deeply explores the role of these zones in the process of rock burst occurrence in the mining area. Its applications include the following aspects:

Rock burst Risk Caused by the Three Load Zones: In the strata of the Three Load Zones, the ‘SLZ’ zone acts as a static load and affects the base stress of the mining area. The stress generated by the movement of the ‘ILZ’ and ‘DLZ’ zones is the source of the induced rock burst. The static load applied by the ‘ILZ’ zone to the coal rib and the dynamic load generated by the periodic movement of the ‘ILZ’ zone both influence the rock burst risk. The delayed loading effect of the ‘DLZ’ zone will affect the stress state of the roadway in the mining area for a long time after the recovery work is completed. During the transition from the fully suspended roof state to the fully in contact with the goaf state, there is a possibility of a ‘mine earthquake’ occurring, which may then trigger a rock burst. In the ‘DLZ’ zone of the 12,305 working face at 118 m, multiple layers of thick hard fine sandstone and sandstone-mudstone are present, and when these critical layers fracture, the working face faces the risk of ‘mine earthquakes’ and rock burst triggered by ‘mine earthquakes’.

Monitoring and Early Warning of the Movement State of the Three Load Zones: Using the Three Load Zones model, the influence range of the forward support pressure and lateral support pressure in the ‘ILZ’ zone can be calculated. This range should be a key area for mine pressure monitoring during the recovery process of the working face, and the density of stress meter placement in this area should be appropriately increased. If there are permanent roadways in the forward support pressure influence range and lateral support pressure influence range of the ‘DLZ’ zone, a stress monitoring system that can meet long-term monitoring tasks should be installed.

For the movement of the ‘ILZ’ and ‘DLZ’ zones, especially the suspended roof state of the ‘DLZ’ zone, microseismic systems can be used for monitoring. According to the positioning results of the microseismic monitoring, microseismic events in the height range of the ‘ILZ’ zone indicate that the strata in the ‘ILZ’ zone are moving. The monitoring results can be used to assess the suspended roof state and predict roof loading events. Microseismic events in the height range of the ‘DLZ’ zone indicate that the strata in the ‘DLZ’ zone are moving. Combined with the borehole column data, when the fracture height reaches the position of the hard strata, preventive measures for ‘mine earthquakes’ should be implemented.

Rock burst Prevention Design Based on the Three Load Zones Structure: To meet the requirements of rock burst prevention, corresponding prevention designs should be made for the strike direction and dip direction based on the strata quality and stress influence range of the Three Load Zones.

In the strike direction, the layer-by-layer movement of the overlying strata can provide dynamic early warning of roof loading time and cycle, so that adequate rock burst prevention preparations can be made before roof loading, preventing rock burst occurrences. The mining advance speed determines the movement parameters of the ‘ILZ’ zone and the magnitude of the dynamic stress. For working faces with good strata quality in the ‘ILZ’ zone, dynamic pressure is larger during initial and periodic loading, so a slower mining speed should be selected based on on-site conditions to reduce the suspended roof length in the ‘ILZ’ zone, thereby alleviating dynamic pressure and reducing the rock burst risk.

In the dip direction, the movement patterns of the overlying strata can predict high-stress areas around the working face, guiding and adjusting the development design and mining plans of adjacent drifts or recovery working faces. By avoiding stress concentration areas, the likelihood of rock burst can be reduced. The height and thickness of the Three Load Zones strata determine the influence range of forward support pressure and lateral support pressure. During mining design, for rock burst prevention purposes, the horizontal position of roadways should be reasonably designed to avoid the regions of peak stress transmission. The design using narrow coal pillars or large working face mining can fundamentally reduce the rock burst risk in working face roadways.

For roadways already in a high-stress state or affected by ‘ILZ’ zone stress, large-diameter drilling for pressure relief should be conducted before recovery. By using appropriate drilling parameters, stress can be transferred to the side walls of the roadway, converting the instantaneous rock burst instability into slow, large deformation damage, thereby preventing rock burst. For roadways affected by ‘DLZ’ zone stress, stress monitoring systems should be used to continuously and repeatedly conduct large-diameter drilling for pressure relief, maintaining low-stress, low-density conditions in the coal and rock body throughout the use period of the roadway to ensure the safety of personnel and equipment.

To enhance the model’s adaptability to special geological conditions, we will propose the following modifications: (1) For ultra-thick hard strata (e.g., gabbro): An equivalent thickness conversion method will be introduced, incorporating a stiffness coefficient to adjust the DLZ zone thickness; (2) For weak/fractured roof strata: A dynamic adjustment model for the ILZ zone will be established based on the RQD index. (3) Next-phase research: Machine learning-based intelligent inversion of Three-Zone parameters will be developed to further improve the model’s applicability in complex geological settings. These refinements will significantly strengthen the model’s practical utility in engineering applications.

The research findings of this paper can be widely applied to coal mines with rock burst risks, providing technical support for the prevention and control of rock burst disasters. The research results can also be formulated into technical specifications for rock burst prevention and promoted to other similar coal mines nationwide.

### Conclusion

Mining activities will cause a redistribution of the support pressure in the working face. The distribution pattern and peak pressure are influenced by the movement of the overlying strata. Understanding the distribution pattern of the support pressure in the working face is of great significance for rock burst risk assessment. This paper primarily investigates the relationship between overlying strata structure and mining-induced stress, and establishes a structural model of the overlying strata that includes mining depth and mining-induced factors, leading to the following conclusions:

1. Based on the relationship between the boundary conditions of the mining area and the overlying strata structure, and according to the different loading methods of the strata, the ‘Three Load Zones’ overlying strata structure model was proposed to describe the impact on the mining area’s rock burst stress field. The overlying strata in the mining area are divided into the ‘Instantaneous Load Zone’ (ILZ), ‘Delayed Load Zone’ (DLZ), and ‘Static Load Zone’ (SLZ), and their respective ranges are defined.
2. Based on the movement patterns of the Three Load Zones during the recovery process, a model for estimating the stress influence range of the Three Load Zones was established. The forward support pressure and lateral support pressure exerted on the coal rib by the ‘ILZ’ and ‘DLZ’ zones during the recovery process were calculated, and the distribution patterns of these pressures were analyzed. The dynamic changes in the Three Load Zones and their impact on rock burst risk in the mining area were also examined.
3. Taking Kongzhuang Coal Mine as a case study, the roof failure and forward support pressure distribution of the 7433 goaf mining face were studied. The reliability of the Three Load Zones theory in calculating the peak support stress and influence range was verified by combining microseismic monitoring results and numerical calculation results. By integrating the actual case of a mine in Shandong, the application value of the ‘Three Load Zones’ in rock burst risk evaluation, rock burst early warning monitoring, and the prevention and control of rock burst was discussed.

## Data Availability

The data that support the findings of this study are available from the corresponding author upon reasonable request. Requests for access to these data should be made to Quanjie ZHU (zhqj2016@ncist.edu.cn).
